# Epigenetic regulation in hematopoiesis and its implications in the targeted therapy of hematologic malignancies

**DOI:** 10.1038/s41392-023-01342-6

**Published:** 2023-02-17

**Authors:** Ailin Zhao, Hui Zhou, Jinrong Yang, Meng Li, Ting Niu

**Affiliations:** Department of Hematology, West China Hospital, Sichuan University, 610041 Chengdu, Sichuan China

**Keywords:** Haematological cancer, Cancer genetics

## Abstract

Hematologic malignancies are one of the most common cancers, and the incidence has been rising in recent decades. The clinical and molecular features of hematologic malignancies are highly heterogenous, and some hematologic malignancies are incurable, challenging the treatment, and prognosis of the patients. However, hematopoiesis and oncogenesis of hematologic malignancies are profoundly affected by epigenetic regulation. Studies have found that methylation-related mutations, abnormal methylation profiles of DNA, and abnormal histone deacetylase expression are recurrent in leukemia and lymphoma. Furthermore, the hypomethylating agents and histone deacetylase inhibitors are effective to treat acute myeloid leukemia and T-cell lymphomas, indicating that epigenetic regulation is indispensable to hematologic oncogenesis. Epigenetic regulation mainly includes DNA modifications, histone modifications, and noncoding RNA-mediated targeting, and regulates various DNA-based processes. This review presents the role of writers, readers, and erasers of DNA methylation and histone methylation, and acetylation in hematologic malignancies. In addition, this review provides the influence of microRNAs and long noncoding RNAs on hematologic malignancies. Furthermore, the implication of epigenetic regulation in targeted treatment is discussed. This review comprehensively presents the change and function of each epigenetic regulator in normal and oncogenic hematopoiesis and provides innovative epigenetic-targeted treatment in clinical practice.

## Introduction

Hematologic malignancies are among the most common cancers and can involve all systems and organs. Hematologic malignancies mainly include leukemia, lymphoma, and multiple myeloma (MM), all of which are highly heterogenous in molecular characteristics, leading to severe difficulties in individualized treatment. Moreover, hematologic malignancies, especially MM, are often incurable and finally relapse or become refractory, appealing for innovative treatment strategies to increase treatment response and to improve their prognosis. Recent studies found that hematologic malignancies show recurrent methylation-related mutations, abnormal methylation profiles of DNA, and abnormal histone deacetylase expression, especially in leukemia and lymphoma. Furthermore, the hypomethylating agents and histone deacetylase inhibitors are effective to treat acute myeloid leukemia and T-cell lymphomas, indicating that epigenetic regulation is indispensable to hematologic oncogenesis. Thus, a comprehensive exploration of the change and the influence of each epigenetic mediators on hematologic oncogenesis assists to develop an innovative targeted treatment to improve the treatment response and prognosis of the patients.

**Fig. 1:**
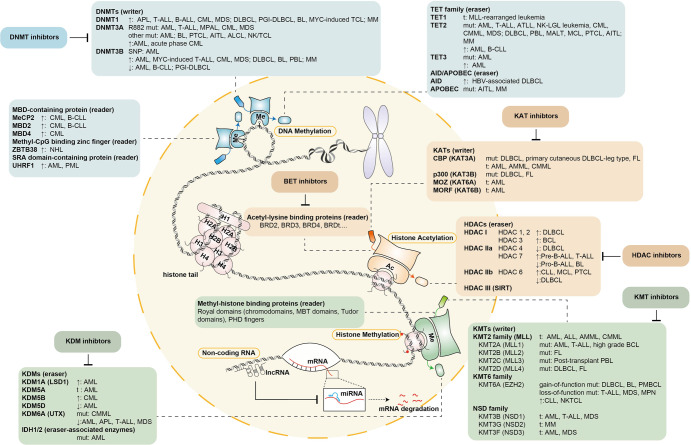
The overview of epigenetic regulation of hematologic malignancies. Epigenetic regulation of hematologic malignancies mainly includes DNA methylation, histone acetylation, histone methylation, and noncoding RNA. First, DNA methylation depends on writers, readers, and erasers. DNMTs are writers of DNA methylation, and targeted treatments focusing on DNMTs have been developed. Readers of DNA methylation include MBD-containing proteins, methyl-CpG binding zinc fingers, and SRA domain-containing proteins. Erasers of DNA methylation mainly consist of the TET family and the AID/APOBEC family. Second, histone acetylation depends on the writer KATs, the reader acetyl-lysine binding proteins, and the eraser HDACs. Correspondingly, innovative treatments targeting KATs, BETs, and HDACs are being developed. Third, histone methylation depends on the writer KMTs, the reader methyl-histone binding proteins, and the eraser KDMs. KMT inhibitors and KDM inhibitors are being explored in the treatment of hematologic malignancies. In addition, microRNAs and long noncoding RNAs also contribute to hematologic oncogenesis

Epigenetics originally referred to heritable features of a cellular phenotype that were independent of changes in DNA sequence. With the development of numerous studies and enlightened perspectives, epigenetics currently defines chromatin-based reactions that regulate DNA-templated processes. Chromatin consists of DNA and histones in a macromolecular complex that serves as a scaffold for genome packing. Histones are mainly divided into five categories. H2A, H2B, H3, and H4 are highly conserved, and an octamer consisting of two of each wrapped with 146 base pairs of DNA serves as the core of nucleosomes. H1 is variable among species and binds with internucleosome linear DNA to form a higher-level structure. According to the composition of chromatin, epigenetic regulation mainly includes alterations in DNA modifications, alterations in histone modifications, chromatin remodeling, and noncoding RNAs (ncRNAs) (Fig. 1). Epigenetic regulation plays an important role in various DNA-based processes, including DNA replication, repair, and transcription. With the development of epigenetic techniques, studies have explored the role of epigenetic regulation in hematopoiesis and have depicted the epigenome in hematologic malignancies. This review aims to highlight the influence of DNA modifications, histone modifications, and ncRNAs in hematopoiesis and their implications in targeted therapy of hematologic malignancies.

## DNA methylation alteration is common in hematologic malignancies

DNA methylation was first discovered from pneumococcal types when DNA was discovered as the hereditary material in mammals.^1,2^ DNA methylation is characterized by the carbon-5 position of the cytosine adding with a methyl group to form 5-methylcytosine (5mC).^3^ DNA methylation usually happens in the cytosine-guanine dinucleotides (CpG) sites by the DNA methyltransferase (DNMT) enzymes.^3^

### DNA methylation in the normal genome

In the human genome, CpG sites spread over the whole genome, and are highly methylated, except CpG islands in normal somatic cells.^4–7^ Approximately 45% of the mammalian intergenic regions of the genome consist of transposable elements and viral elements, which are silenced by heavy methylation.^8^ However, CpG islands, consisting of 500–2000 bps GC-rich sequences, are usually unmethylated in normal cells.^4,5,9^ In addition, around 70% of gene promoters include CpG islands.^10^ While most CpG islands are unmethylated, many oncogenes have methylated promoter CpG islands in normal cells, and the function of oncogenes were repressed.^11^ In addition, distal regulatory regions, like enhancers, lack CpG islands and are regions with low methylation.^12^

### DNA methylation in the overall cancer cells

However, normal epigenetic processes are disrupted in multiple diseases, including inflammatory diseases, precancerous conditions, and cancers.^13–16^ Generally, overall genome-wide hypomethylation or hypermethylation with regional DNA hypermethylation or hypomethylation of CpG islands can be observed in these diseases.^13,17,18^ A loss of DNA methylation of transposable elements and repeat elements, accompanied by aberrant expression, results in the dysregulation of pathways.^19^ The hypermethylation of unmethylated promoter CpG islands is related to the inhibition of tumor suppressor genes and functional genes. However, the hypomethylation of methylated promoter CpG islands is related to the activation of oncogenes, while CpG-poor regions in CpG island shores or enhancers tend to undergo methylation in cancer cells.^20,21^

### The change of DNA methylation in hematologic malignancies

Extensive studies have reported that DNA methylation patterns in regulatory regions play an important role in the development of cell proliferation and the function of leukemic stem cells (LSCs). The change of DNA methylation in hematologic malignancies is frequently observed in two aspects, the methylation level of methyltransferase genes themselves, and the mutations of methyltransferase genes or demethylase genes. Regarding the former aspect, studies have shown that the promoter of *DNMT1* gene was unmethylated in all acute promyelocytic leukemia (APL) patients.^22^ Tirdad et al. found that patients with B and T-acute lymphoblastic leukemia (ALL) had unmethylated promoters in the *DNMT1* gene, whereas the control group showed a relatively methylated promoter.^23^ Abnormal hypomethylation of the *DNMT3A* gene was also observed in 55.3% of acute myeloid leukemia (AML) patients, and was related to an adverse prognosis in cytogenetically normal (CN)-AML patients.^24^ The hypomethylation of promoters or intragenic regions of methyltransferase genes probably led to DNMT hyperactivation, causing cancer cell hypermethylation in leukemia. In terms of the latter aspect, recurrent *DNMT3A* mutations and *TET2* mutations were found in leukemia. For example, patients with *DNMT3A* mutations and *IDH1/2* mutations exhibited a mixed DNA hydroxy-/methylation profile compared with samples from healthy controls.^25^ AML patients with *DNMT3A* mutations displayed lower levels of DNA methylation as well as fewer concurrently hypermethylated genes.^26^ A loss of *DNMT3A* caused enhancer hypomethylation in *FLT3*-ITD-associated leukemias and was critical for the inhibition of leukemic transformation.^27^ Studies identified somatic mutations by exome sequencing in AML-M5, and found that *DNMT3A* mutants caused decreased enzymatic activity and abnormal affinity to histone H3.^28^ In chronic myelomonocytic leukemia (CMML) patients with *TET2* mutations, AIM2 and SP140, the non-CpG island promoters, were hypermethylated.^29^ *TET2* was found to be transcriptionally repressed or silenced in 71% and 17% of T-ALL patients, respectively.^30^ Apart from the frequently observed DNA methylation alterations, the change of other methylation mediators was also discovered. For example, the high expression of the methyltransferase DNMT3B was related to poor prognosis in AML patients.^31^ Myelodysplastic syndrome (MDS) and AML-mesenchymal stem cells (MSCs) displayed global hypomethylation and underexpression of the methyltransferase DNMT1 and the methyl-binding protein UHRF1.^32^ Changes in the methylation patterns of methyl-binding proteins, MBD2 and MeCP2, were observed in B-chronic lymphocytic leukemia (CLL).^33^ The recurrent change in DNA methylation profile in AML and ALL could predict patients’ prognosis and treatment efficacy.^34^

### Enzymes of DNA methylation

Various enzymes participate in DNA methylation, and can be divided into three categories based on their function. The proteins that catalyze and cause a certain modification are referred to as writers. The proteins that eliminate an existing modification are referred to as erasers. The proteins that recognize an existing modification and recruit other macromolecular complexes to the template chain are referred to as readers.

#### Writers: DNA methyltransferases directly catalyze DNA methylation

**Fig. 2:**
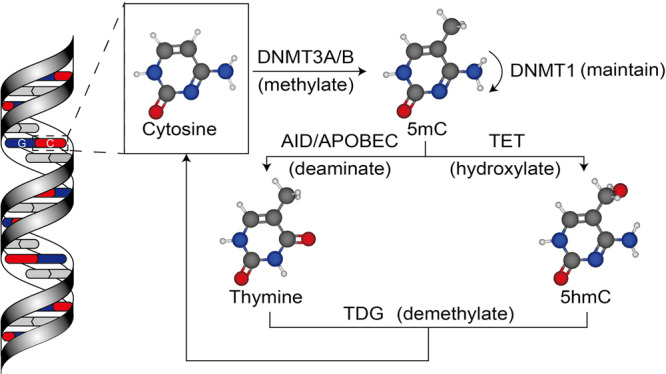
The methylation and demethylation pathways. DNMT3A and DNMT3B can methylate the cytosine (5mC) and set up a new methylation pattern to unmodified DNA. DNMT1 maintains an established pattern of DNA methylation during DNA replication. 5mC can be chemically modified at two sites: the amine group and the methyl group. The amine group of 5mC can be deaminated by AID/APOBEC to thymine (Thy). The methyl group of 5mC can be added a hydroxyl group by TET enzymes to generate 5-hydroxymethyl-cytosine (5hmC). Eventually, the products, Thy and 5hmU, can be recognized and removed by TDG

The DNMT family can directly catalyze DNA methylation, including DNMT1, DNMT3A, and DNMT3B.^35^ It has been known for many years that DNMT3A and DNMT3B, so-called de novo DNMTs, can develop a new methylation pattern in unmodified DNA.^36,37^ The structure and function of DNMT3A and DNMT3B are extremely similar. On the other hand, DNMT1 primarily methylates hemimethylated DNA,^38,39^ and can repair DNA methylation.^40^ In addition, DNMT1 can maintain an established pattern of DNA methylation, copying the DNA methylation pattern when DNA replicates (Fig. 2).^41–43^ The three DNMTs are necessary for embryonic or neonatal development.^37,44^ DNMT3A is also critical for hematopoietic stem cell (HSC) differentiation.^45^ DNMT2 and DNMT3L have no catalytic function. DNMT2 functions as an RNA methyltransferase. DNMT3L is associated with DNMT3A and DNMT3B, and promotes their methyltransferase activity.^46–48^

#### DNMT1 maintains the methylation

DNMT1 maintains an established pattern of DNA methylation. Upregulated DNMT1 could induce abnormal regional hypermethylation, and cause the pathogenesis of leukemia. The phenomena of DNMT1 alteration have been frequently observed in leukemia and lymphoma. Studies have shown that DNMT1 is overexpressed in APL,^22^ AML,^49,50^ ALL,^50^ MDS, and chronic myelogenous leukemia (CML) patients.^49,51^ The methylation of CpG islands in promoter regions is often observed in lymphomas. *DNMT1* was overexpressed in Burkitt’s lymphoma (BL),^52^ diffuse large B-cell lymphoma (DLBCL),^53^ primary gastrointestinal diffuse large B-cell lymphoma (PGI-DLBCL)^54^ and other lymphomas.^55,56^ *DNMT1* overexpression was identified as an independent risk factor in PGI-DLBCL.^54^ Furthermore, *DNMT1* overexpression was associated with advanced clinical stages and resistance to treatment in DLBCL.^53^ DNMT1 could predict survival and Ki-67 expression in DLBCL patients received with the R-CHOP regimen.^57^ In addition, the expression of *DNMT1* in U266 myeloma cells was higher than that in normal control cells.^58^

The function of DNMT1 in hematological malignancy progression has been reported in different pathways. In leukemia, conditional knockout of *DNMT1* inhibited leukemia development, and *DNMT1* haploinsufficiency delayed the progression of leukemogenesis and impaired LSC self-renewal without altering normal hematopoiesis.^59^ In AML, Wang et al. discovered that the expression level of *DNMT1* in AML patients was decreased compared with that in healthy controls, and was negatively regulated by miR-148a in AML cell lines.^60^ In ALL, recent studies demonstrated that the level of exosomal *DNMT1* mRNA transcripts was elevated in ALL patients, which might reprogram leukemia progression.^61^ In CML, DNMT1 was upregulated by BCR-ABLp210, and promoted tumor stem cell priming,^62^ and silencing the *DNMT1* gene could inhibit the proliferation and promote the apoptosis of CML K562 cells.^63^ Regarding lymphoma, DNMT1 played a critical role in the maintenance of MYC-induced T-cell lymphomas and BL and contributed to abnormal methylation.^64,65^ In addition, DNMT1 was correlated with cell cycle and DNA replication gene sets in DLBCL.^66^ In MM, DNMT1 also promoted the methylation of SOCS-1^67^ and TJP1^68^ in myeloma cells, thereby decreasing their expression and regulating MM development.

#### DNMT3A catalyzes de novo methylation

Among the different DNMTs, the DNMT3A has been investigated and reported the most frequently in hematologic malignancies, and its change is presented below in the order of leukemia, lymphoma, and MM, respectively.

In leukemia, mutations of *DNMT3A*, especially in R882H, are one of the most frequent recurrent genetic changes in AML. Mutations of *DNMT3A* also occur in clonal hematopoiesis, MDS, ALL, CMML, mixed phenotype acute leukemia (MPAL), and pediatric AML patients.^69–80^ The prevalence of *DNMT3A* mutations was 7.4% in adult Thai AML patients,^81^ 25% in US patients,^82^ 19.7% in Korean patients,^83^ 4.0–13.9% in Chinese patients,^84,85^ 6% in Brazilian patients,^86^ 20.9% in German patients^87^ and 17.9% in Egyptian AML patients.^88^ The majority of *DNMT3A* mutations have been reported to be related to increased relapse^89^ and poor survival in AML,^82,83,90–100^ CMML,^101^ ALL,^71,75,102,103^ Chinese pediatric AML,^70^ and Chinese pediatric ALL patients.^71^ In addition, DNMT3A1 and DNMT3A2V have been reported as the main variants in AML.^104^ In contrast to de novo AML, most mutations occurred in the methyltransferase domain other than arginine at position 882 in therapy-related and secondary AML.^105^

Furthermore, DNMT3A mutations were associated with several clinical characteristics in leukemia. Studies showed that *DNMT3A* mutation was not only related to the intermediate-risk cytogenetic group in de novo AML^90,106,107^ but also associated with age; the white blood cell (WBC) count, platelet (PLT) count, and blast percentage in peripheral blood; M4/M5 immunophenotype; *FLT3* mutation; *NPM1* mutation; *IDH1/2* mutation; and *CEBPA* mutation in AML.^87,92,97,98,107–112^ In T-ALL, *DNMT3A* mutation was also associated with increased age, high WBC, high BM blast cell percentage, and extramedullary disease.^103^ Regarding cell of origin, the *DNMT3A* ^R882H^ mutation occurred more frequently in the T-ALL subtype than in the B-ALL subtype.^71^ *DNMT3A* mutations also showed a marked predilection for T-lineage differentiation in MPAL.^93^ Besides the genetic mutation of *DNMT3A*, overexpression of the DNMT3A protein was also reported in AML and the acute phase of CML.^49,113^ The expression levels in AML patients were higher than ALL patients or healthy controls,^65,114^ and DNMT3A expression acted as a potential prognostic biomarker.^113^

In addition, *DNMT3A* mutations, together with other mutations, were related to the prognosis of leukemia patients. For example, *FLT3* and/or *NPM1* mutations contributed to survival differences in *DNMT3A*-mutant patients.^81,115^ However, the *IDH1/2* gene had little effect on patients’ survival with a *DNMT3A* mutation.^116^ Dose escalation of anthracycline in the induction regimen was correlated with improved survival in AML patients with *DNMT3A* mutations.^117^ Allogeneic hematopoietic stem cell transplantation (allo-HSCT) could increase the survival of CN-AML patients with *DNMT3A* mutations.^118^ In patients with complete response (CR) after complete donor chimerism allo-HSCT, no *DNMT3A* ^R882H^ mutation was found.^119^ *DNMT3A* mutant could always be detected during remission and did not predict prognosis in AML patients.^119–122^ While, *DNMT3A* ^R882^/*FLT3*-ITD had poor prognosis in AML patients after allo-HSCT.^123–125^ *DNMT3A* variants were also associated with the progression of CML after tyrosine kinase inhibitor (TKI) therapy.^126^

The relationship between the type of *DNMT3A* mutation and clinical outcomes remained controversial in leukemia patients. One study reported that outcomes of patients with R882 and non-R882 missense mutations were similar, while patients with truncation mutations had comparable outcomes to those of patients with wild-type *DNMT3A*.^99^ However, in another study, patients with the R882 mutation and those with non-R882 mutations showed different clinical outcomes; patients with the R882 mutation had unfavorable relapse-free survival (RFS), while patients with non-R882 mutations had favorable overall survival (OS).^87^ In addition, the *DNMT3A*^R882^ mutations were associated with adverse prognoses in older patients, while non-R882 mutations were related to worse prognoses in younger patients.^109^ Mutations in *DNMT3A* exon 23 have also been reported to independently predict an unfavorable prognosis in older AML patients.^127^

Though less frequently, *DNMT3A* mutations were also found in lymphomas. *DNMT3A* mutations occurred in 11% of T-cell lymphomas,^128^ 26.6–39% of peripheral T-cell lymphomas (PTCLs),^129–131^ 26.1–34% of angioimmunoblastic T-cell lymphomas (AITLs),^129,131–133^ and were also found in NK/T-cell lymphoma/leukemia,^134^ breast implant-associated anaplastic large cell lymphoma (ALCL) and BL.^135^ However, *DNMT3A* mutations were infrequent in primary cutaneous CD4^+^ small/medium T-cell lymphoproliferative disorder (PCSMLPD),^136^ monomorphic epitheliotropic intestinal T-cell lymphoma (MEITL) and enteropathy-associated T-cell lymphoma (EATL).^137^ *DNMT3A* mutations were more commonly seen in patients of African ancestry compared than in those of European ancestry.^138^ *DNMT3A* mutations or expression were related to prognosis in lymphoma patients. Study findings suggested that high expression of *DNMT3A* was significantly related to worse OS and progression-free survival (PFS) in patients with PGI-DLBCL patients treated with R-CHOP regimen.^54^ *DNMT3A* mutation was found to be correlated with shorter PFS in AITL patients.^133^ Since *DNMT3A* mutations were significantly decreased after therapy,^139^ they could serve as sensitive indicators in circulating tumor DNA (ctDNA) and provide a noninvasive method of monitoring minimal residual disease in AITL.^140^

The evidence for the effects of DNMT3A on MM is contradictory. Study findings indicated that *DNMT3A* mutations were present in newly diagnosed MM patients and those treated by autologous stem cell transplantation (ASCT).^141,142^ Furthermore, *DNMT3A* overexpression was demonstrated in the cells of MM patients.^58,143^ Different from lymphoma, studies showed that *DNMT3A* underexpression was associated with worse OS in MM patients.^144^

DNMT3A potentiated hematologic oncogenesis in many ways. First, the *DNMT3A* ^R882^ mutation stimulated the mTOR pathway^145^ and reactivated the leukemic transcription factor MEIS1,^146^ which initiated AML. *DNMT3A* ^R882^ mutations also enhanced abnormal stem cell gene expression, which promoted leukemia progression.^147^ Studies also found that *DNMT3A* ^R882^ mutations reduced AML cell apoptosis by augmenting PRDX2,^148^ and induced CML by disturbing DNA methylation.^149^ In T-cell lymphomas, the *DNMT3A* mutation was found in both programmed cell death 1 (PD-1) ^+^ T cells and supportive cells, indicating its role in both tumor cells and microenvironment.^150,151^ Second, DNMT3A was found to be a haploinsufficient tumor suppressor in various hematologic malignancies. The haploinsufficiency of DNMT3A could transform *FLT3*-ITD myeloproliferative neoplasm (MPN) into AML,^152^ and cooperate with oncogenic *KRAS* to promote the development of T-ALL.^153^ The haploinsufficient tumor suppressor effect of DNMT3A was also reported in CLL,^154^ CD8^+^ PTCL,^155^, and AITL.^156–159^ Third, *DNMT3A* mutations were closely related to treatment response. Studies showed that *DNMT3A* mutations induced anthracycline resistance in AML by impairing nucleosome remodeling,^160^ and evaded chemotherapy and infiltrated the central nervous system in a patient with AML.^161^ *DNMT3A*^R882H^-dependent AML cells were sensitive to hypomethylating agents, such as azacytidine (AZA)^162^ and decitabine.^163^ In addition, DOT1L could be a therapeutic target,^164^ and resistin may serve as an ancillary drug for AML patients with *DNMT3A* mutation.^165^

#### *DNMT3B* also catalyzes de novo methylation

*DNMT3B* caused the generation of abnormal methylation during the development of hematologic malignancies. In leukemia, studies showed that *DNMT3B* was overexpressed in MDS, AML and CML patients.^49,51,65^ The *DNMT3A* expression level in AML patients was higher compared with ALL patients.^65^ However, another study found that *DNMT3B* expression was decreased in AML^113^ and B-CLL.^33^ Exploring the polymorphism C46359T in the *DNMT3B* promoter, Li et al. found different distributions of genotypes in different races and that the CT heterozygote was related to the pathogenesis of AL.^166^ Furthermore, *DNMT3B* mutations, polymorphisms, and expression were related to AML prognosis. *DNMT3B* overexpression was related to adverse prognosis in older CN-AML patients.^167^ The G allele of rs1569686 in *DNMT3B* represented poor outcomes for AML, while the C allele of rs2424908 was associated with favorable outcomes.^168,169^

In lymphoma, *DNMT3B* expression was also increased in BL,^52^ DLBCL^53^, and plasmablastic lymphoma (PBL),^170^ and it was identified as an adverse prognostic factor.^53^ *DNMT3B* overexpression was associated with advanced clinical stages and resistance to treatment in DLBCL.^53^ However, studies also found that the expression of *DNMT3B* was lower in PGI-DLBCL patients.^54^ In MM, the expression of *DNMT3B* was increased in in U266 and RPMI8226 myeloma cells.^58,171^

DNMT3B mainly affected the progression of hematologic malignancies. Some studies found that the loss of *DNMT3B* accelerated MLL-AF9 leukemia progression.^172^ Others showed that high expression of *DNMT3B* contributed to abnormal DNA methylation and MYC-driven tumor development in T-ALL and BL.^65^

#### Readers: the methyl-binding proteins recognize DNA methylation and mediate subsequent reactions

DNA methylation can directly inhibit the binding of transcription factor and repress gene transcription when it occurred at the regulatory region of a gene.^173^ In addition, the methylated site recruits methyl-binding proteins (MBPs), the readers, and then attracts the members of the chromatin remodeling complex, to activate or inhibit transcription.^174,175^ MBPs mainly include three separate families of proteins: “methyl-CpG-binding domain (MBD)-containing proteins”, “methyl-CpG binding zinc fingers”, and “Set and RING-associated (SRA) domain-containing proteins”.^176^

##### MBD-containing proteins

This is the first identified MBP family, of which all members have the conserved MBD domains. MeCP2 was the first MBP to be identified.^177^ MeCP2 had an MBD domain, which comprises 70–85 amino acids, and recognizes and binds to methylated sites.^178^ Then, this MBD domain could recognize other proteins with methyl-binding potentials.^179^ According to the presence of domains excepted MBD, there were three groups of this family: (1) MeCP2-MBDs: MeCP2, MBD1, MBD2, MBD3, MBD4, MBD5, and MBD6; (2) histone methyltransferase-MBDs: SETDB1 and SETDB2; and (3) histone acetyltransferase-MBDs: BAZ2A and BAZ2B. However, the classification above is according to their structural characteristics other than methyl-binding abilities, thus not all proteins can bind to methylated DNA. The MBD domain of MeCP2,^180^ MBD1,^181^ MBD2^179^, and MBD4^182^ can bind to methylated CpG. However, MBD3,^183^ MBD5, MBD6,^184^ BAZ2A, and BAZ2B^185^ cannot bind to methylated DNA. Moreover, both SETDB1 and SETDB2 play a role as protein lysine methyltransferase activity.^186,187^

The expression of MBD2 and MeCP2 was found to be increased in CML.^188^ Studies found that deregulation of the epigenetic repertoire, including MBD2 and MeCP2 in B-CLL.^33^ The homozygous CC genotype in *MBD2* (rs603097, -2176C>T) was associated with favorable outcomes of non-Hodgkin lymphoma (NHL).^189^ MeCP2 could epigenetically regulate SOCS5 expression in T-ALL and the SPAN-XB core promoter sequence in MM.^190,191^ The MeCP2/SIN3a deacetylating complex could epigenetically silence the proapoptotic gene *BIM* in ALCL.^192^ The knockdown of *MBD2* inhibited apoptosis induced by B1 and overcame the resistance caused by Bcl-2 in HL60 cells.^193^ *MBD4* was found to be overexpressed in imatinib-resistant K562 cells, and the knockdown of *MBD4* expression decreased cell survival after treatment with hydrogen peroxide and doxorubicin.^194^

##### Methyl-CpG binding zinc fingers

The second family of MBPs is the “methyl-CpG binding zinc fingers” family, the members of which, including Kaiso, zinc-finger, and BTB domain containing 4 (ZBTB4), and ZBTB38. They can identify methylated and unmethylated DNA by zinc-finger motifs.^195^ The first member of the family was Kaiso.^196^ ZBTB4 and ZBTB38 are homologs of Kaiso proteins.^197^ Kaiso is a transcription factor and the binding partner of p120-catenin in the cytoplasm.^198^ It is encoded by the *ZBTB33* gene which is located on the X chromosome.^199^ Kaiso binds to a couple of methylated CpG dinucleotides.^196^ ZTBT4 and ZBTB38 can bind to a single methylated CpG.^197^

The knockdown of *Kaiso* increased cell proliferation and blocked granulocytic differentiation in CML blast crisis.^200^ Kaiso protected human umbilical vein endothelial cells against apoptosis through the overexpression of *BCL2* and decreased expression of *BAX* and *BIK*.^201^ HBV preferential target genes, such as *ZBTB38*, showed significantly altered expression levels in NHL.^202^

##### SRA domain-containing proteins

The third family of MBPs is the “SRA domain-containing proteins” family, including UHRF1 and UHRF2. They contain the SRA domain, which can bind to methylated DNA. The SRA domain usually bind to the hemimethylated regions,^203,204^ while the MBD/MeCP2 domains preferentially bind to the paired methylated DNA. UHRF1, also known as ICBP90 (human) or Np95 (mouse), binds to hemimethylated DNA and then attracts DNMT1 to methylate the sequence during replication.^205,206^ UHRF2, also known as NIRF or Np97, is involved in the regulation of the cell cycle.^207^

The *UHRF1* level was found to be increased in aneuploid AML.^208^ The *UHRF1* promoter was found to be hypomethylated in leukemia patients.^209^ *UHRF1* was found to regulate the transcriptional repressor HBP1 through MIF in T-ALL, and *UHRF1*-knockdown mice lived longer.^210^ Furthermore, difluoromethylornithine (DFMO) and thymoquinone were found to downregulate *UHRF1* in Jurkat cells.^211,212^ UHRF1 promoted ubiquitination-mediated degradation of the tumor-suppressive promyelocytic leukemia protein.^213^ Berberine was a novel drug for the treatment of MM via targeting *UHRF1*.^202^

#### Erasers: DNA demethylases remove the existing methylation

DNA demethylation is a dynamic process including passive or active pathways. Passive DNA demethylation happened in dividing cells, where newly incorporated cytosine remains unmethylated by DNMT1 inhibition or dysfunction. Active DNA demethylation can happen in both dividing and nondividing cells, which requires enzymes to remove 5mC to develop a permissive state for subsequent gene expression.^214–217^ Enzymes in active DNA demethylation involve the ten-eleven translocation (TET) methylcytosine dioxygenases, activation-induced cytidine deaminase/apolipoprotein B mRNA-editing enzyme complex (AID/APOBEC), and thymine DNA glycosylase (TDG).^218–220^ 5mC can be modified at two sites, the amine group and the methyl group. The AID/APOBEC can deaminate an amine group of 5mC to thymine (Thy).^221^ The TET enzymes can add a hydroxyl group to the methyl group of 5mC and generate 5-hydroxymethyl cytosine (5hmC). IDH enzymes converted isocitrate to α-ketoglutarate, which is critical for TET catalytic function. Eventually, the products, Thy and 5hmC, can be recognized and removed by TDG (Fig. 2).^221^

#### *TET1*: oxidation of 5mC to 5-hydroxymethyl cytosine (5hmC)

The disruption of normal DNA demethylation was identified to be associated with oncogenesis. Both genetic mutations and abnormal protein expression of the TET family were reported in hematologic malignancies. *TET1* was found to act as oncogene in MLL-rearranged leukemia.^222^ *TET1* expression was decreased, whereas the expression of *TET2* and *TET3* was increased in AML.^223,224^ In refractory AML, *TET1* expression was increased compared to that in treatment-responsive patients.^225^ Moreover, *TET1*, *TET2*, and *TET3* overexpression was found to be related to poor prognosis in AML.^223,224,226^ The expression of *TET1* and *TET3* decreased in CLL, and high *TET2* expression was related to longer survival in CLL.^227^ In addition, *TET* mutations could also be observed in hematological malignancies. The most frequently mutated gene was *TET2*, with *TET2* mutations happening in 32% (10/31) of patients with human T-cell lymphotropic virus type I (HTLV-I)-induced acute adult T-cell leukemia (ATL).^228^ *TET2* mutations also occurred in AML and T-ALL patients.^229,230^ In the experimental study, *TET1* promoted the growth of T-ALL and could be antagonized via PARP inhibition.^231^

#### *TET2*: oxidation of 5mC to 5-hydroxymethyl cytosine (5hmC)

*TET2* mutations were found to happen in patients with hematological malignancies, and affected DNA hypermethylation.^232^ In leukemia, studies found that 33% of patients had low levels of *TET2*-specific differentially methylated CpG (DMC) methylation, and these patients had longer OS.^233^ *TET2* mutations occurred in 6–23% of CN-AMLs,^234–236^ 1.4% of childhood AMLs,^76^ 7.6% of younger adult AMLs,^237^ and 11.8–27.4% of AMLs^82,235,238–245^ and were unfavorable prognostic factors.^82,236,238,239,241,246^ *TET2* mutations occurred in 14% of adult T-cell leukemia/lymphomas (ATLLs)^247^ and 32% of ATLs,^228^ and lower *TET2* expression was associated with adult T-cell leukemia aggressiveness.^248^ Furthermore, *TET2* mutations were also observed in 28% of chronic natural killer large granular lymphocyte (NK-LGL) leukemia cases.^249^ In addition, *TET2* mutations were also observed in advanced phase CML,^250,251^ and the presence of *TET2* SNP rs3442524 suggested disease progression.^251^ However, some studies reported TET2 mutation did not affect prognosis. *TET2* mutation alone was found to have no prognostic impact on event-free survival (EFS) or OS in one study.^245^ Similarly, *TET2* mutations were detected in 19.8–23.3% of secondary AMLs, and did not influence OS.^252,253^ In addition, *TET2* mutations occurred in 32–65% of CMML and were favorable prognostic factors,^29,254–260^ but were associated with poor survival in the presence of *ASXL1* mutations.^261^ While in another study, *TET2* mutations were correlated with poor survival.^262^ There are different types of TET2 mutations, and patients have different clinical outcomes. Complete deletion of the TET_JBP domain (ΔJBP) of TET2 in AML patients had a lower CR rate and shorter EFS and OS.^263^ *TET2* ^I1762V^ acted as a favorable prognostic factor in AML.^264^ *TET2* SNP rs2454206 correlated with improved survival in childhood CN-AML.^265^ *TET2* exon 2 splicing status might improve survival in CN-AML patients.^266^ *TET2* mutations were reported to be associated with several clinical characteristics. *TET2* was associated with a normal karyotype, high WBC count, low PLT count and high age.^238,255,267^ Arginase 1 overexpression was related to *DNMT3A* and *TET2* mutations in lower-grade MDS and CMML.^268^ Besides the genetic alteration of *TET2*, the expression of TET2 protein was also different between patients with hematological malignancies and healthy people. For example, the expression of *TET2* was showed to be increased in B-CLL,^269^ but decreased in AML^270,271^ and childhood ALL,^272,273^ and was related to poor survival.^270,271^

*TET2* is a well-established tumor suppressor in the context of lymphomas. Studies have detected *TET2* mutations in mantle cell lymphoma (MCL),^274^ DLBCL,^275^, and non-anaplastic PTCLs.^276^ *TET2* mutations occurred in 18–92% of AITLs,^129,132,247,277^ 5.7–12% of DLBCLs,^247,278^ 14% of ATLLs,^247^ 11% of PBLs,^279^ 38–64% of PTCLs,^280,281^ 75% of PTCLs with T-follicular helper phenotype (PTCL-TFH),^129^ 85% of mucosa-associated lymphoid tissue (MALT) lymphomas,^282^ and 18% of T-cell lymphomas.^128^ *TET2* mutations were correlated with poor survival in AITL and PTCL, not otherwise specified (PTCL-NOS) patients.^281,283^ Germline *TET2* loss of function was found to cause childhood immunodeficiency and lymphomagenesis.^284^ Besides the genetic alterations, TET expression was found to increase in NHL.^285^ Moreover, *TET2* mutations were correlated with advanced-stage disease, thrombocytopenia, and high International Prognostic Index (IPI) scores in AITL and PTCL-NOS.^281^ *TET2* mutations were related to positive B symptoms.^280^ In MM, *TET2* mutations were also detected,^141,142,286–288^ and might be related to survival and resistance to therapy at relapse.^287^

The recovery of *TET2* function blocked abnormal self-renewal and progression of leukemia.^289^ *MEG3* promoter hypermethylation might result from decreased *TET2* activity in AML.^290^ *TET2* mutations affected DNA methylation in non-CpG island of enhancers and transcription factor-binding sites in CMML.^291^ Moreover, the expression of *TET2* decreased, and promoter methylation of *TET2* significantly increased the risk of ALL.^292^ Cooperation between *KDM6B* overexpression and *TET2* deficiency was found to initiate the pathogenesis of CMML.^293^ *RHOA* mutations were correlated with a T-follicular helper (TFH) cell phenotype and *TET2* and *IDH2* mutations.^294^ *AID* and *TET2* cooperation modulated FANCA expression through active demethylation in DLBCL.^295^ Reduced *TET2* function led to T-cell lymphoma with TFH-like features in mice.^296^

#### *TET3*: oxidation of 5mC to 5-hydroxymethyl cytosine (5hmC)

*TET3* mutations are infrequently observed in AML, but the expression of *TET3* in HSCs and human peripheral blood T cells has been found to decrease with age.^297,298^ The expression of *TET3* was found to be increased in AML, which was associated with poor OS and disease-free survival (DFS).^223,224,227^ *TET3* mutation could be detected in newly diagnosed AML patients.^299^

#### *AID/APOBEC*: deamination of 5mC to thymine (Thy)

The AID/APOBEC family of proteins was a group of cytidine deaminases, and played roles in multiple physiological functions.^300,301^ The family consists of eleven members in humans: AID and APOBEC1, APOBEC2, seven APOBEC3 proteins (APOBEC3A, APOBEC3B, APOBEC3C, APOBEC3D, APOBEC3F, APOBEC3G, APOBEC3H) and APOBEC4.^302–307^ APOBEC1 is hardly expressed in CLL, while APOBEC3A and APOBEC3G are both present in CLL and normal B cells.^308^ In a patient with two relapses, the interplay of aberrant AID/APOBEC activity was observed.^309^ APOBEC overactivity was also observed in primary plasma cell leukemia (pPCL).^310^

Furthermore, aberrant AID/APOBEC activity-associated mutations were enriched in early clonal hematopoiesis-associated mutations in AITL/PTCL-NOS.^311^ AID/APOBEC overactivity was also observed in HBV-associated DLBCL.^312^

APOBEC-associated mutations were enriched in patients with disease progression and were associated with a shorter time to progression in smoldering MM (SMM).^313–315^ APOBEC mutations were detected in MM^316–319^ and might be related to high risk, a shorter progression time, and therapy resistance.^287,317,320^ The *APOBEC* mutation was observed in 3.8% of myeloma cases and was associated with the translocation-mediated deregulation of MAF and MAFB, which was associated with poor prognosis.^319^ Dysregulated APOBEC3G was found to cause DNA damage and promoted genomic instability in MM.^321^ APOBEC3B overexpression was found to constitutively generate DNA substitutions and deletions in myeloma cells.^322^

#### *TDG:* demethylation of Thy and 5hmC to cytosine

TDG was a member of the uracil DNA glycosylase (UDG) superfamily. TDG is required for embryonic development.^323^ DNA hypermethylation of TDG in MM cell lines was found to lead to low expression of genes, and inhibit DNA repair activity during hydrogen peroxide-induced DNA damage.^324^ Moreover, SUMO-1 modification and colocalization with the promyelocytic leukemia protein were required in the noncovalent SUMO-1 binding activity of TDG.^325^

#### *IDH1/2*: conversion of isocitric acid to α-ketoglutaric acid

Isocitrate dehydrogenase (IDH) converted isocitrate to α-ketoglutarate (α-KG). The IDH family included IDH1 and IDH2, and their mutant forms transformed α-KG into 2-hydroxyglutarate (2-HG). 2-HG inhibited DNA demethylases and histone demethylases, thus increasing methylation of both DNA and histones.^326^ There are three conserved mutational hotspots in the IDH enzymes. The mutational hotspot of *IDH1* is R132, while the mutational hotspot of *IDH2* were R140 and R172.

*IDH1* mutations occurred in 6–9% of AML patients, especially in patients with normal karyotype AML (8–16%).^327–333^ *IDH1* mutations often co-occurred with normal karyotypes and *NPM1* mutations,^328–331,333^ and were associated with wild-type *CEBPA* and no *FLT3* mutation.^331^ Published results on the prognostic effects of *IDH1* mutations failed to reach an agreement. Although some studies have shown that *IDH* mutations have no prognostic effect on OS in *IDH*-mutated (*IDH1* and *IDH2*) patients or in overall patients,^328–331^ *IDH1* mutations have a poor prognosis impact on low-risk or intermediate -risk subgroups of patients with normal karyotype AML.^328,331,333^ In AML patients under 60 years old in the low-risk group, 5-year DFS (42% vs. 59%) and OS rates (50% vs. 63%) of *IDH*-mutated patients were significantly lower than those in *IDH* wild-type patients. In low-risk AML patients, *IDH* mutations (combined *IDH1* and *IDH2*) were correlated with lower 5-year RFS and OS rates, respectively.^333^ The same phenomenon was found in *IDH1* or *IDH2* mutations alone, although the number of patients in each subgroup was small and only the RFS analysis was statistically different.^333^ *IDH1* mutations were also correlated with poor EFS and OS in patients with intermediate-risk normal karyotype AML.^328^ *IDH2* mutations have been observed in 8–12% of AML patients,^327–329,333,334^ with a higher mutation frequency of 19% in the normal karyotype.^331^ In almost all cases, *IDH2* and *IDH1* mutations were mutually exclusive.^328,329,331^ *IDH2* mutation could occur at R172 or R140, while R140 mutation happened more frequently.^331,333,334^ Interestingly, *IDH2*^R172^ mutations appeared to repel *NPM1* mutations and *FLT3*-ITD.^331,333,334^ The prognostic effects of *IDH2* mutations were also inconsistent. Some studies reported no prognostic value of *IDH2* mutations,^328,329,333^ while others reported a good prognosis for *IDH2*-mutated patients.^327,334^ In one study, *IDH2* mutations were associated with poor prognosis in a subpopulation of AML patients with normal karyotype and other low-risk factors.^333^ However, *IDH2*^R140^ mutations were correlated with increased survival in all patients as well as in a subgroup of patients with the low-risk group.^327^ In the latter subgroup, patients with *IDH1* or *IDH2* mutations had a significant increase in OS at 3 years compared to patients with *NPM1* mutations without *FLT3*-ITD and patients without *IDH1* or *IDH2* mutations. These results suggested that in normal karyotype AML patients without *FLT3*-ITD, *NPM1* mutations conferred survival benefits only when *IDH* mutations were present simultaneously.^327^ The conflicting results of these studies required further investigation.

Inhibition of *IDH1* mutant promoted cycling of LSCs.^335^ A genomic analysis was performed in R/R AML patients with *IDH* mutations who received ivosidenib (IDH1 inhibitor) treatment, and the results suggested that primary resistance to ivosidenib was correlated with RTK pathway mutations.^336,337^ *IDH2* mutation cooperated with a *NUP98-HOXD13* fusion caused early immature thymocyte precursor ALL.^338^

*IDH2*^R172^ mutation defined a unique subgroup of patients with AITL.^158^ Patients with *IDH2* mutations had increased H3K27me3 and DNA hypermethylation of gene promoters. In a retrospective multicenter study, *IDH2*^R172^ mutation was found in 19–45% AITL patients and was correlated with poor survival,^339^ and different pathologic and clinical features.^132,133,340–342^ When using plasma cell-free DNA as liquid biopsy for the diagnosis of AITL, *IDH2*^R172^ hotspot mutations were detected in 15% of patients.^343^ Studies found that *IDH2* mutation was confined in the malignant T cell of AITL, and that 2-HG was elevated in tumor tissue and serum of patients.^344^

*IDH2* was observed overexpressed in CD138^+^ cells from MM, and it promoted progression and poor prognosis of MM by regulating m6A RNA methylation.^345^ *IDH2* inhibition increased efficacy of proteasome inhibitor in MM, MCL, and BL cells.^346^

## Histone modifications mainly include acetylation, methylation, and phosphorylation

### Histone modifications in normal hematopoiesis

Posttranslational modifications of amino acids, especially lysines, at the N-terminal tail of the histone core change the chromatin configuration, ranging from the condensed repressive status to the open active status. There are at least sixteen types of histone modifications, including acetylation, methylation, phosphorylation, ubiquitylation, sumoylation, and ADP ribosylation. The histone modifications most essential for hematopoiesis and hematologic malignancy oncogenesis are acetylation, methylation, and phosphorylation.

HSCs are commonly recognized to arise from the endothelial-to-hematopoietic transition, which occurs at the early arterial endothelial cell, hemogenic endothelial cell, pre-HSC, and long-term HSC stages. Based on the results of low-input itChIP-seq and Hi-C assays, the stimulative histone modifications, H3K27-ac and H3K4me1, which regulate enhancer activation, already exist in the initiation stage of early arterial endothelial cells and hemogenic endothelial cells. During the differentiation of HSCs, the stimulative H3K27-ac gradually increases while the repressive H3K27me3 decreases, leading to the steady expression of HSC genes.^347^ In lineage differentiation, histone modification also plays an important role. A crucial lineage-mediating protein, BAP1, regulates the balance between lymphopoiesis and myelopoiesis by altering the histone modifications of the promoters of pro-hematopoietic factors and myelopoiesis-promoting factors. Upon BAP1 deficiency, H2AK119-ub1 and H3K27me3 are strengthened on the promoter of the pro-hematopoietic factor Scf, leading to its downregulation. In addition, H3K4-me3 and H3K27-ac are strengthened on the promoter of the myelopoiesis-promoting factor Csf3, leading to its upregulation and steering the differentiation to myeloid lineage, which causes CMML-like disease.^348,349^ The dynamic balance among the writers, readers, and erasers of histone modifications is essential to hematopoiesis and oncogenesis, which will be discussed below (Fig. 1).

### The change of histone acetylation pattern in hematologic malignancies

Acetylation reduces the positive charge of lysines and in turn weakens the interaction between histones and negatively charged DNA, causing an open active euchromatin status, as evidenced by ChIP-seq, which reveals histone acetylation at promoters and enhancers of actively transcribed genes.^350^ Histone deacetylase (HDAC) erases the acetyl group from lysines in the N-terminus, which amplifies the positive charge of lysines and in turn strengthens the interaction between histones and the negatively charged DNA, leading to the condensed repressive heterochromation status.^351^ Histone lysine acetyltransferase (KAT, also named HAT) and HDAC counteract subtly to sustain the hematopoiesis balance. In normal erythropoiesis, erythroid-stimulating transcription factors, such as GATA-1, recruit KAT to the β-globin locus to increase the acetylation levels of H3 and H4, which in turn upregulates globin during erythropoiesis.^352^ When the expression of wild-type or chromosomal-fused transcription factors is abnormal due to epigenetic dysregulation, the hematopoiesis balance is disturbed and malignancies occur.^353^ For example, TAL1/SCL is a wild-type transcription factor steering differentiation to the erythroid and megakaryocytic lineage instead of the myeloid lineage. Upon association with the coactivating complex of p300 and PCAF, TAL1/SCL was found to be upregulated and propelled erythropoiesis leading to murine erythroleukemia. However, TAL1/SCL was found to be downregulated upon association with the corepressive complex of HDAC1 and mSin3A, impeding erythropoiesis.^354^ Regarding fusion proteins caused by chromosome translocation, *PML-RARα, RUNXI-MTG8*, and *AML-ETO1* recruit abnormal histone-modifying enzymes to target genes, causing leukemogenesis.^355^ In lymphoma, various oncogenic mutations which were related with histone acetylation were also discovered.^356^

#### Writers: histone acetyltransferases directly catalyze histone acetylation

KATs are divided into two types. Type A KATs are mainly nuclear and consist of five families, the p300/CBP, MYST, GNAT, general transcription factor/orphan KAT, and SRC/NCoA families. Type B KATs, consisting of KAT1, are mainly cytoplasmic.^357^ Of the many KAT families, the p300/CBP family, and MYST family are the most essential KATs in hematologic malignancy oncogenesis, and are presented below, respectively.

The p300/CBP family includes p300, also named KAT3B or EP300, and CBP, also named KAT3A or CREBBP. CBP has been found to bind to viral oncoprotein E1A, which is associated with oncogenesis.^358^ CBP could also serve as a tumor suppressor, as various loss-of-function mutations have been found in lymphoma. For example, monoallelic mutations in *CBP*, which inactivated KAT and led to B-cell oncogenesis, were discovered in 40% of follicular lymphoma (FL) patients, up to 30% of DLBCL patients, and 26% of primary cutaneous DLBCL-leg type patients.^359,360^ In addition, these inactivating mutations were found to cooperate with BCL2, BCL6, and MYC to promote lymphoma development, promoting the aggressiveness of lymphoma and indicating its indispensable role as tumor suppressors.^361^ The chromosomal translocation of MLL-CBP, t(11;16)(q23;p13), was found to specifically drive the proliferation of granulocyte/macrophage progenitors (GMPs), leading to acute myelomonocytic leukemia (AMML) and CMML.^362^ Furthermore, *P300* mutations were also found in 10% of FL and DLBCL patients,^363^ which disrupted the binding of transcription factors, such as CREB and c-Myb, and caused multilineage hematopoietic defects.^364^ In DLBCL, mutations of CBP/p300 could repress acetylation of H3K27, stimulate the Notch pathway, which in turn upregulated CCL2/CSF1, leading to M2 polarization of the tumor-associated macrophages and lymphoma progression.^365^

The MYST family includes MOZ, MORF, HBO1, TIP60, and HMOF. This family was frequently discovered to promote leukemogenesis by translocation, which consistently activated the downstream signaling pathway. For example, MOZ, also named KAT6A, was discovered to translocate with various genes. *MOZ-CBP* translocation, t(8;16)(p11;p13), led to consistent activation of KAT and was related to M4/M5 AML.^366^ *MOZ-TIF2* translocation, induced by inv(8)(p11;q13), simulated aggressive leukemia by introducing stem cell activity to progenitor cells, leading to persistent self-renewal and leukemogenesis.^367^ MORF, also known as KAT6B, whose translocation with CBP induced by t(10;16)(q22;p13) caused a loss of monoacetylated H4K16, is a well-recognized indicator of AML transformation.^352^ HBO1, also known as KAT7, is the acetyltransferase of H3K14, and its loss leads to abnormal consumption and exhaustion of HSCs; therefore, HBO1 is indispensable for HSC maintenance and self-renewal.^368^

#### Readers: acetyl-lysine binding proteins recognize histone acetylation and mediate subsequent reactions

Readers of histone lysine acetylation include a highly conserved binding domain, the bromodomain. One of the most explored and targeted readers is the BET family, consisting of BRD2, BRD3, BRD4, and BRDt. However, few studies have revealed the contribution of BET family genetic alterations to the oncogenesis of hematologic malignancies. Most of the studies exploring its pathogenic influence have focused on solid tumors, for example, midline carcinoma. The translocation of the nuclear protein of testis (NUT) to BRD4, induced by t(15;19)(q14;p13.1), impeded the differentiation and promoted the proliferation of epithelial cells. On the one hand, the fusion protein stimulated p300 and amplified hyperacetylation, which recruited BRD4 and oncogenic transcription components. On the other hand, aberrant occupation of BRD4 inhibited c-fos transcription, which in turn inhibited epithelial differentiation.^369^ A similar oncogenic translocation to NUT was also discovered in BRD3.^370,371^ The evidence of histone acetylation readers contributing to hematologic malignancies are lacking and requires further investigation.

#### Erasers: histone deacetylases remove the existing acetylation of histones

HDACs are categorized into four classes, including the zinc-dependent classic HDACs (class I, II, and IV) and the NAD^+^-dependent nonclassic HDACs (class III). The three categories of classic HDACs can be divided based on sequence homology to yeast deacetylases. Class I HDACs, including HDAC1, HDAC2, HDAC3, and HDAC8, are homologous to reduced potassium dependency-3 (Rpd3) and localize mainly in the nucleus; these HDACs form multiprotein complexes and regulate transcription and proliferation. Class II HDACs are homologous to histone deacetylase-1 (Hda1). Class IIa HDACs, including HDAC4, HDAC5, HDAC7, and HDAC9, move between the nucleus and cytoplasm. Class IIa HDACs have only one catalytic domain and have weaker deacetylation capability than the nucleus-localizing class IIb HDACs, including HDAC6 and HDAC10, which contain two catalytic domains. Class IV HDACs share a homologous sequence to both Rpd3 and Hda1 and reside in the nucleus. Class IV HDACs include only HDAC11, which is the smallest among the various HDACs. The nonclassic class III HDACs consist of SIRT1 to 7.

The change of HDAC manifested itself mainly through altered protein expression, instead of genetic mutations, in hematologic malignancies. Impaired expression of H3K4ac1 and H3K4ac3 was commonly observed in lymphoma.^372^ The overexpression of HDAC1 and HDAC2 was discovered in DLBCL compared to their expression in normal lymphoid tissues.^373^ The inhibition of HDAC1 and HDAC2 was found to impede Eμ-myc B-cell lymphoma development via apoptosis stimulation and proliferation repression, indicating the oncogenic role of HDAC1 and HDAC2.^374^ HDAC1 expression was found to be significantly related to worse prognosis in DLBCL patients and PTCL-NOS patients.^375^ HDAC3 overexpression was pathogenic when CBP mutations were present. In normal differentiation, the BCL6-SMRT-HDAC3 complex counteracts CBP to regulate the acetylation balance of H3K27. However, upon CBP malfunction due to mutations, the BCL6-SMRT-HDAC3 complex overacts in enhancer deacetylation of the B-cell signaling pathway without opponents, leading to B-cell lymphomagenesis.^376^ In contrast, HDAC4 was a tumor suppressor. It serves as a corepressor of BCL6 and represses oncogene expression upon recruitment, impeding B-cell lymphomagenesis.^377^ DLBCL patients with high HDAC2 expression and low HDAC4 expression were found to display significantly worse prognosis than those with low HDAC2 expression and high HDAC4 expression, also indicating the pathogenic role of HDAC2 and the protective role of HDAC4 in DLBCL.^378^ HDAC6 expression varies in different malignancies, and its role is controversial. In DLBCL, HDAC6 showed a tendency toward low or negative expression in most patients, and the minority of DLBCL patients who showed strong HDAC6 expression had a significantly better prognosis than the others. However, in PTCL, HDAC6 showed a tendency toward to be overexpressed, which was significantly related to worse prognosis.^373^ Apart from PTCL, HDAC6 was also found to play a pathogenic role in MCL, and its inhibition exerted an antitumor influence.^379^ HDAC6 could also regulate immunogenicity and downregulate PD-1/PD-L1 in cell subpopulations of CLL and MM.^380,381^ HDAC6 inhibition could decrease Treg cells and myeloid-derived suppressor cells (MDSCs) and increase the cytotoxicity of T cells, strengthening immune response of the host to counteract myeloma cells.^381^ In a CLL Eµ-TCL1 mice model, HDAC6 inhibition could reduce the immunosuppression caused by tumor T cells, and enhance immunomodulation of the supportive B cells,^380^ indicating the potential combined treatment of HDAC inhibitors and immune treatment, which is presented in the Targeted Therapy section of this review. HDAC7 was crucial to committing pro-B cells to pre-B cells by interacting with MEF2C. Thus, HDAC7 downregulation was found in pro-B-ALL and BL, while its upregulation was found in pre-B-ALL and B-ALL t(8;14).^382,383^ HDAC7 was also shown to be essential to the survival and TCR engagement of thymic T cells during differentiation, whose overexpression was discovered in T-ALL patients.^384^

The class III HDACs are the SIRT family, which has been found less associated with HDAC function. The SIRT family showed a tendency to regulate HSC aging through various pathways. For example, SIRT1 preserved aged HSCs by stimulating FOXP3 and inhibiting the mTOR pathway. Moreover, SIRT1 rendered HSCs vulnerable to stepwise mutation development in CML, while SIRT1 inhibition strengthened DNA repair and improved the sensitivity of leukemic stem cells (LSCs) to imatinib.^385^ The evidence of the SIRT family affecting hematologic malignancies is relatively limited. Further investigation is needed to better reveal their relationship.

### The change of histone methylation pattern in hematologic malignancies

The methylation of histones mainly occurs on lysines and arginines. Lysine methylation manifests itself as mono-, di-, and trimethylation. Arginine methylation can be symmetrical or asymmetrical. Unlike acetylation, which alters chromatin status and regulates transcription by changing the charge of the lysine, methylation does not change the charge of lysine or arginine. Whether lysine methylation stimulates or inhibits transcription depends on the position and features of methylation. For example, methylation on H3K4, H3K36, and H3K79 is more often related to active euchromatin status, while methylation on H3K9, H3K27, and H4K20 usually causes repressive heterochromatin.^386^ Moreover, H3K9me1 is commonly seen in actively transcribed genes, while H3K9me3 is frequently discovered in repressed genes.^387^ Arginine methylation is commonly related to gene activation.^388^

#### Writers: histone methyltransferases directly catalyze methylation of the histone

Histone methyltransferases are mainly divided into lysine methyltransferases (KMTs) and protein arginine methyltransferases (PRMTs). KMTs mainly consist of KMT2, KMT6, NSD, and KMT1 families. Members of the KMT2 family, including KMT2A (also known as MLL1), KMT2B (also known as MLL2), KMT2C (also known as MLL3), KMT2D (also known as MLL4), KMT2F (also known as SETD1A), and KMT2G (also known as SETD1B), catalyze the methylation of H3K4. MLL is essential for proliferation and lineage differentiation during hematopoiesis. MLL can not only activate genes by methylating H3K4 and recruiting KAT but also inhibit genes by recruiting HDACs and polycomb group (PcG) proteins.^389^ Recurrent mutations and fusion proteins related to MLL have been reported in various hematologic malignancies. On the one hand, KMT2A mutations have been reported in AML (2.5%), T-ALL (5.6%), and high-grade B-cell lymphoma (14%).^390^ Recurrent *KMT2B* mutations were discovered in more than 90% of FLs.^391^ *KMT2C* mutations were revealed in 45.5% of posttransplant plasmablastic lymphomas.^392^ *KMT2D* mutations were reported in more than 70% of FL patients and nearly 30% of DLBCL patients, which impaired its methylation capability and steered the transformation of cancer stem cells.^391,393,394^ On the other hand, *MLL* fusion proteins caused by the translocation of chromosome 11q23 and more than 60 counterparts have been discovered in leukemia, such as the t(9;11) translocation and the t(4;11) translocation identified in de novo AML and ALL, and the t(11;16) translocation discovered in therapy-related AMML and CMML.^362^ In *MLL* fusion proteins, DNA binding domains were found to be preserved, and *HOX* genes were overexpressed and promoted leukemogenesis.^395^ Moreover, partial tandem duplication (PTD) of *MLL* was also found in AML patients with normal karyotypes, which occurred after DNA methylation-related mutations (*TET2*, *DNMT3A*, *IDH1*/*2*) but before kinase mutations (*FLT3*, *RAS*).^396^ *MLL*-PTD was found to be significantly related to worse prognoses in AML patients.^327^

The KMT6 family includes KMT6A, also named EZH2, which catalyzes the trimethylation of H3K27. EZH2 serves as an important part of the polycomb repressive complex (PRC), which negatively regulates transcription. EZH2 has a dual role, as evidenced by its oncogenic role and tumor-suppressive role in different hematologic malignancies. As oncogenes, EZH2 gain-of-function mutations (Y641, A682, and A692) in catalytic SET domains amplified H3K27 methylation, which in turn inhibited the differentiation of plasma cells, promoting oncogenesis in B-cell lymphoma.^363,397^ These mutations were discovered in 22% of DLBCLs, 10% of BLs, and 4% of primary mediastinal B-cell lymphomas. Excessive EZH2 expression was also found to exist in NK/T-cell lymphoma and CLL.^398,399^ As tumor suppressors, loss-of-function mutations of EZH2 were found in T-ALL, MDS, and MPN.^400–402^

Members of the NSD family, including KMT3A (also known as SETD2), KMT3B (also known as NSD1), KMT3G (also known as NSD2), and KMT3F (also known as NSD3), catalyze the methylation of H3K36 and H4K20. *NSD1* was found to be fused to *NUP98*, a part of the nuclear core complex, caused by the translocation of t(5;11)(q35;p15.5), in 16.1% of pediatric AML with normal cytogenetics.^403^ The *NUP98*/*NSD1* translocation was significantly related to *FLT3*-ITD variations and worse prognoses, which could be attenuated only through bone marrow transplantation.^404,405^ NUP98/NSD1 protein was shown to influence CD34^+^CD133^+^ hematopoietic precursor cells in MDS, AML, and T-ALL patients; transform bone marrow cells; and trigger AML in mouse models.^406,407^ *NSD2* was found to be fused to *FGFR3*, caused by the translocation of t(4;14)(p16.3;q32), in 15% of MM patients. The *NSD2*/*FGFR3* translocation was significantly related to 13q- and worse prognoses, which could not be overcome even by a high-dose regimen and ASCT.^408,409^ *NSD3* was also found to fuse to *NUP98*, caused by the t(8;11)(p11.2;p15) translocation, in AML and radiation-related MDS patients.^410,411^

PRMT, catalyzing the mono- and demethylation of histone arginine, participates in the epigenetic regulation of leukemogenesis. PRMT4 was found to be indispensable to the onset of *MLL*-*AF9*-driven AML.^412^ PRMT4 could also negatively regulate CBP/p300 coactivation by methylating CBP.^413^ PRMT5 stimulated the self-renewal and viability of LSCs by triggering the Wnt/β-catenin pathway in CML.^414^ PRMT7 was shown to regulate glycine metabolism to preserve LSCs in CML, and the loss of PRMT7 downregulated glycine decarboxylase and propelled glycine metabolism to produce toxic methylglyoxal in LSCs without influencing normal hematopoiesis. PRMT7 inhibition impeded leukemogenesis based on CML mouse models and primary CD34^+^ cells from CML patients.^415^

#### Readers: methyl-histone binding proteins recognize histone methylation and mediate subsequent reactions

Since the methylation of lysines occurs in various sites in different forms, the readers of methylated lysines are diverse. Methyllysine readers are mainly divided into two groups based on recognition domains, the Royal domain (chromodomains, MBT domains, and Tudor domains) and PHD fingers. In *NUP98*-*PHD* (*PHF23* or *JARID1A*) fusion AML, the PHD finger was found to read H3K4me2/3 and inhibit its removal at various lineage differentiation transcription factors, which caused persistent activation of Hox, Pbx1, and Gata3 transcription factors and led to leukemogenesis. Mutations in PHD fingers, which prevented H3K4me2/3 reading, were shown to impede leukemogenesis, indicating the essential role of methyl reading in leukemogenesis.^416^ In addition to hematologic malignancies, abnormal methyllysine reading was more commonly seen in solid cancers. HP1 belongs to the chromodomain family, and decreased HP1 expression was reported in breast cancer, colon cancer, ovarian cancer, and papillary thyroid carcinoma.^417^ ING belongs to the PHD finger family, and its mutations were discovered in melanoma.^418^ Further research is required to explore the influence of altered methylation readers on hematologic malignancies.

#### Erasers: histone demethylases remove the existing methylation of histones

The most explored lysine demethylases (KDMs) can be divided into two groups. The first group requires an amine oxidation reaction relying on flavin adenine dinucleotide (FAD) as cofactor, which can only demethylate mono- or dimethyllysine. The second group, the Jumonji demethylase, relies on oxidation and radical attack, such as a-ketoglutarate, and can methylate mono-, di-, and trimethyllysine.

LSD1, also named KDM1A, belongs to the first group. LSD1 has a dual role, as evidenced by its transcription-activating and repressing ability. On the one hand, LSD1 was found to demethylate H3K9me1/2 and repress transcription when related to androgen or estrogen receptors.^419,420^ On the other hand, LSD1 was shown to promote transcription after demethylating H3K4me1/2 in promoter regions. LSD1 has also been shown to serve as an essential member of transcription repressing complexes, such as CoREST, HDAC2, and ZNF217.^421^ Moreover, LSD1 was found to demethylate H3K4 after HDAC deacetylation, which in turn assisted in and amplified transcription inhibition, as evidenced by the impaired LSD1 function caused by HDAC inhibitors.^422,423^ In hematopoiesis, LSD1 was found to mediate the function of the TAL1, GATA-1, and C/EBPa transcription factors; steer erythroid differentiation,^424,425^ and promote erythroleukemia by inhibiting GFI1 superenhancers.^426^ LSD1 inhibition was demonstrated to reactivate PU.1-dependent enhancers and eradicate AML in mouse models.^427^

The KDM5 family, which demethylates H3K4, belongs to the second group and consists of KDM5A, KDM5B, KDM5C, and KDM5D. *KDM5A* translocation with *NUP98* was frequently found to be pathogenic in AML patients, and *KMD5A* downregulation suppressed proliferation and induced the apoptosis of AML cells.^428,429^ The loss or inhibition of *KDM5D* promoted cell differentiation, impeded the growth of APL cells, and improved sensitivity to all-trans retinoic acid treatment.^430^ *KDM5B* was overexpressed in CML, and mediated myeloid differentiation and Toll-like receptors via GATA and AP-1 transcription factors. *KDM5B* knockdown impaired colony formation in CML cells.^431^ *KDM5D*, located on chromosome Y, encodes a demethylase of H3K4. *KDM5D* downregulation was associated with human and mouse AML. The contribution of mosaic loss of chromosome Y to leukemogenesis might be attributable to *KDM5D* loss.

KDM6A, also named UTX, plays various roles in hematopoiesis and hematologic malignancies. UTX is essential for protecting young hematopoietic stem progenitor cells (HSPCs) from aging. *UTX* deficiency was associated with the aggregation of reactive oxygen species, reduced DNA damage repair, and aging in HSPCs.^432^ Regarding hematologic malignancies, *UTX* mutations were found in 8% of CMML patients.^74^ *UTX* has been demonstrated to be a tumor suppressor that represses myeloid leukemogenesis and preserves drug sensitivity in MDS, AML, APL, and even T-ALL.^433–437^ However, studies also discovered that *UTX* served as a pro-oncogenic cofactor indispensable to leukemia development in TAL1-positive T-ALL, and UTX inhibition significantly impeded TAL1-positive leukemia.^438^

Because methylation is essential to both DNA modification and histone modification, the 2-HG caused by *IDH* mutants inhibited demethylases of both DNA and histones, especially the Jumonji family. Recurrent *IDH1*/*2* mutations were found in 20% of AML patients, and these mutations transformed isocitrate into 2-HG instead of the original a-KG in the tricarboxylic acid cycle, leading to the accumulation of 2-HG and a decrease in a-KG. However, the Jumonji family relies on a-KG to exert a demethylating function, and 2-HG displays a similar orientation in the catalytic core of the JmjC domain, leading to the repression of Jumonji family function, which in turn increases the histone methylation level. The phenomena and influence of *IDH* mutations on hematologic malignancies have been discussed in the DNA methylation section of this review. IDH inhibitors have been approved by the Food and Drug Administration (FDA) for AML treatment and are presented in the Targeted therapy section below.^439^

### The change of histone phosphorylation is less frequently reported in hematologic malignancies

Histone phosphorylation displays less evidence than histone acetylation and methylation in hematologic malignancies. Histone phosphorylation plays a role in crucial cellular reactions, including apoptosis, transcription, DNA repair and replication, and usually occurs on threonine, tyrosine, and serine residues. Kinases not only stimulate signal transduction but also phosphorylate histones. For example, *JAK2* mutations are frequently discovered in MPN, ALL, and AML. H3Y41 phosphorylated by *JAK2* was found to disturb the binding of the chromatin repressor HP1a and to stimulate the Lmo2 oncogene to promote leukemogenesis.^355^ In primary mediastinal B-cell lymphoma and Hodgkin lymphoma (HL), excessive IL-13 and the amplification of chromosome 9p24 were found to stimulate JAK2, which in turn phosphorylated H3Y41 and activated various oncoproteins, including *MYC* and *JAK2* itself. Moreover, *JAK2* was found to stimulate PD-L1/2 expression, which conferred immune escape of cancer cells.^440–442^ Furthermore, in activated B-cell-like (ABC) DLBCL, JAK1 was discovered to phosphorylate H3Y41, which in turn stimulated MYC, MYD88, and IRF4. *MYD88* overexpression was shown to produce excessive IL-6 and IL-10 which in turn activated JAK1 in a positive feedback loop.^443^ In CML, H2AX phosphorylation was found to be stimulated by imatinib and resveratrol, which in turn triggered apoptosis via the caspase-3/Mst1 pathway.^444–446^

## Noncoding RNAs mainly include micrornas and long noncoding RNAs to exert epigenetic regulation function

Traditionally, ncRNAs refer to RNA transcripts that do not encode proteins.^447,448^ Although around 75% of the human genome can be transcribed, only about 2% of it is translated into mRNAs that encode proteins.^449–451^ A substantial percentage of the human genome is translated into regulatory, catalytic, and structural RNAs.^447,451–453^ Recent studies have demonstrated that some ncRNA transcripts can also encode small peptides within 100 amino acids.^454^ NcRNAs participate in a variety of cellular activities, regulate gene expression and protein function, and are functionally involved in normal development, physiological functions, and the pathogenesis of illness. On the basis of RNA length, many kinds of ncRNAs can be distinguished. Currently, the most extensively researched ncRNAs are microRNAs (miRNAs) and long noncoding RNAs (lncRNAs).It has been revealed that the dysregulation of miRNAs and lncRNAs is involved in all hallmarks of cancer initiation and progression,^455–457^ including hematologic malignancies (Fig. 1).^458^ Table 1 shows the selected miRNAs and lncRNAs implicated in hematologic malignancies.

**Table 1:** Selected microRNAs and long noncoding RNAs involved in hematologic malignancies

Name	NcRNA Class	Implicated hematologic malignances	Function roles in tumorigeneses	References
miR-155	miRNA	DLBCL(ABC), CLL, HL, PMBL, PTLD, pediatric BL, CTCL, AML	Oncogene	^484,731–736^
miR-150	miRNA	CLL, MDS, MLL-associated leukemia	Oncogene	^737–739^
miR-21	miRNA	CLL, DLBCL(ABC)	Oncogene	^491,733^
miR-221	miRNA	DLBCL(ABC)	Oncogene	^740^
miR130b	miRNA	DLBCL	Oncogene	^499^
miR-29	miRNA	CLL	Oncogene	^508,741^
miR-181	miRNA	CLL, AML, APL	Oncogene/tumor suppressor	^508^^,742–745^
miR-15a/16–1	miRNA	CLL, APL, MM	Tumor suppressor	^475,476,733,746^
miR-143/145	miRNA	CLL, DLBCL, MALT, BL	Tumor suppressor	^747^
miRNA-193b-3p	miRNA	T-ALL	Tumor suppressor	^748^
miR-497/195	miRNA	ALL	Tumor suppressor	^749^
miR-22	miRNA	AML	Oncogene	^750,751^
miR-9	miRNA	MLL-rearranged AML/t(8;21), EVI1^+^AML	Oncogene/tumor suppressor	^752–754^
miR-17–92 cluster	miRNA	MLL-rearranged AML	Oncogene	^755,756^
miR-146a	miRNA	del(5q) MDS/MDS-derived AML	Tumor suppressor	^757–760^
miR-125b	miRNA	AML, B-ALL	Oncogene	^761,762^
miR-126	miRNA	AML	Oncogene	^763–765^
miR-155	miRNA	FLT3-ITD-induced AML	Oncogene	^735,736^
193a	miRNA	AML	Tumor suppressor	^766,767^
miR-193b	miRNA	AML	Oncogene/tumor suppressor	^768–770^
miR-223	miRNA	AML	Tumor suppressor	^771,772^
miR-495	miRNA	MLL-rearranged AML	Tumor suppressor	^773^
miR-30–5p	miRNA	MM	Tumor suppressor	^774^
miR-137 and miR-197	miRNA	MM	Tumor suppressor	^775^
miR-214	miRNA	MM	Tumor suppressor	^776^
miR-26a	miRNA	MM	Tumor suppressor	^777^
LUNAR1	lncRNA	T-ALL	Oncogene	^564^
TCLlnc1	lncRNA	T-ALL	Oncogene	^566^
DANCR	lncRNA	AML	Oncogene	^565^
HOXBLINC	lncRNA	NPM1-mutant AML	Oncogene	^778^
HOXB-AS3	lncRNA	NPM1-mutated AML	Oncogene	^779^
BlackMamba	lncRNA	ALK^−^ anaplastic large cell lymphoma	Oncogene	^780^
HOXB-AS3	lncRNA	NPM1-mutated AML	Oncogene	^779^
MEG3	lncRNA	AML	Tumor suppressor	^567^
H19	lncRNA	CML	Tumor suppressor	^781^
BGL3	lncRNA	BCR-ABL-positive CML	Tumor suppressor	^568^
NEAT1	lncRNA	MM	Oncogene	^782^
CRNDE	lncRNA	MM	Oncogene	^783^
MALAT1	lncRNA	MM	Oncogene	^784^

*ABC* activated B-cell-like, *ALL* acute lymphoblastic leukemia, *AML* acute myeloid leukemia, *APL* acute promyelocytic leukemia, *BL* Burkitt’s lymphoma, *CLL* chronic lymphocytic leukemia, *CTCL* cutaneous T-cell lymphoma/leukemia, *DLBCL* diffuse large B-cell lymphoma, *EVI1* Ecotropic viral integration site 1, *FLT3* FMS-like tyrosine kinase 3 receptor, *HL* Hodgkin lymphoma, *ITD* internal tandem duplication, *lncRNA* long noncoding RNA, *MALT* mucosa-associated lymphoid tissue, *MDS* myelodysplastic syndrome, *miRNA* microRNA, *MLL* mixed lineage leukemia, *MM* multiple myeloma, *ncRNA* noncoding RNA, *NPM1* Nnucleophosmin 1, *PMBL* primary mediastinal B-cell lymphoma, *PTLD* posttransplant lymphoproliferative disorder

### The physiologic role of microRNAs

MiRNAs are small single-stranded ncRNAs that are usually approximately 22 nucleotides (nt) in length,^459^ and they were first identified in 1993 in studies on the development of *C. elegans*.^460,461^ MiRNAs regulate posttranscriptional gene expression predominantly via sequence-complementary binding to the 3' untranslated region (3' UTR) of target messenger RNA (mRNA), resulting in the degradation of the associated mRNA or the suppression of protein expression.^462,463^ Moreover, miRNAs can also interact with other targets, such as loci of the protein-coding region of mRNAs,^464–466^ 5’ UTRs of mRNAs,^467^ intronic and intergenic transcripts,^468,469^ and other ncRNAs.^470,471^

The synthesis of miRNAs involves multiple processes. RNA polymerase II (Pol II) first transcribes miRNA-encoding genes in the nucleus, producing a lengthy primary transcript called pri-miRNA. The pri-miRNAs fold back on themselves to create a distinctive hairpin structure, which is recognized and cleaved by the heterotrimeric complex of Drosha endonuclease and its companion protein, DGCR8, resulting in the release of a 60-nt pre-miRNA. The pre-miRNAs are then exported to the cytoplasm by Exportin 5 and the Ran-GTP complex, where Dicer cleaves both pre-miRNA strands near the loop to form a miRNA duplex. One of the miRNA strands is chosen to serve as the guide strand, which is then loaded into an Argonaute protein to form the RNA-induced silencing complex (RISC).^463,472,473^ After the RISC is formed, the miRNA within the RISC pairs with the target mRNA to direct posttranscriptional repression.^463^ A single miRNA can target various genes, while multiple miRNAs can target a single gene.^463^ According to the latest miRbase (v22) data, the total number of human miRNAs identified to date includes 2654 mature miRNA molecules,^474^ and these miRNAs play extensive fundamental roles in normal physiologic processes and disease states.

In 2002, miRNAs' role in cancer was established for the first time, upon discovering frequent deletions and downregulation of the miR15 and miR16 genes in CLL.^475^ miR-15/16 have been identified as negative regulators of the BCL2 oncogene and the receptor kinase-like orphan receptor 1 (ROR1) gene.^476,477^ Subsequently, miRNA dysregulation has been found in almost all studied cancers, including solid tumors and hematologic malignancies.^478–481^ In cancer, miRNAs function mainly in two ways: as tumor suppressors or as oncogenes.^482^ Changes in miRNA function in cancer cells are mainly due to changed expression levels of mature or precursor miRNAs compared to related normal tissues.^457^ The underlying processes of miRNA expression pattern deregulation are diverse, and include miRNA locus deletions or amplifications, miRNA gene mutations, epigenetic and transcriptional regulation, posttranscriptional modification, and dysregulation of miRNA processing.^473^

### MiRNAs can function as oncogenes in hematologic malignancies

MiRNAs can function as oncogenes in hematologic malignancies, thereby contributing to leukemia and lymphoma tumorigenesis. These microRNAs can directly suppress the activity of tumor suppressors or indirectly limit the activity of oncogene-negative regulators.^482^ A typical example is miR155, which is mainly involved in B-cell malignances.^483–485^ The copy number of miR-155 is 10- to 30-fold more in B-cell lymphomas, including DLBCL, than in normal B cells.^484^ Transgenic mice carrying a miR-155 transgene expressed selectively in B cells were found to exhibit a gradual process of an initiation and progression of B-cell malignancy.^483^ Overexpression of miR-155 in lymphoid organs resulting in disseminated lymphoma in a mouse model, whereas withdrawal of miR-155 in tumor-established mice resulted in rapid lymphoma remission.^484^ Furthermore, delivery of anti-miR-155 therapy as the sole treatment intervention was found to effectively suppress tumor growth.^486^ A negative regulator of the kinase AKT, Src homology-2 domain-containing inositol 5-phosphatase 1 (SHIP1) was targeted by MiR-155 and its expression was suppressed. Decreased SHIP1 expression was found to enhance AKT signaling, subsequently promoting cell proliferation and survival and ultimately leading to lymphoma.^485^ Cobomarsen, a miR-155 inhibitor, has been evaluated in multiple types of lymphoma (NCT 02580552). Another example is miR-21, which has been identified as being overexpressed in a variety of solid tumors and hematologic malignancies.^478,487–489^ Overexpression of MiR-21 was observed in CLL,^490^ DLBCL,^491^ AML, and HL.^492,493^ Overexpression of miR-21 in a mouse model was capable of the initiation, maintenance and survival of tumors, ultimately leading to a pre-B-cell lymphoma. Strikingly, the inactivation of miRNA-21 resulted in rapid and completed tumor regression in a few days, induced by the apoptosis and rapid proliferative arrest of tumor cells.^494^ The miR-17–92 cluster has also been demonstrated to be an oncogene. The human miR-17–92 cluster is located on chromosome 13q31.3 and comprises six miRNAs: miR-17, miR-18a, miR-19a, miR-19b-1, miR-20a, and miR-92-1.^495^ The transcript C13orf25, which included the miR-17–92 cluster, was first observed to have elevated expression in malignant lymphoma.^496^ The overexpression of the miR-17–92 cluster, in concert with the expression of the c-myc oncogene, accelerated the formation of B-cell lymphoma tumors in a mouse model, according to a subsequent study.^497^ C-myc might directly bind to the miR17–92 cluster locus and stimulate the miR17–92 cluster’s expression.^498^ However, this cluster of microRNAs inhibited E2F1, a transcription factor that promotes cell cycle progression and is also targeted by c-myc.^498^ In an analysis of a large cohort of 532 newly diagnosed cases of DLBCL, serum levels of the miR130b were correlated with shorter PFS and OS.^499^ Comprehensive research revealed a correlation between serum miR130b and tumor miR130b. In DLBCL, the activity of miR130b on the IFNAR1/p-STAT1 axis reduced lymphoma cell OX40L expression. Through the interaction of OX40L/OX40 lymphoma cells and Th17 cells, the downregulation of tumor cell OX40L expression leads to the increase of Th17 cells at the tumor site.^499^ MiR130b overexpression also promoted the function of Th17 cells by increasing IL17 production. Thus, miR130b contributed to the progression of DLBCL by fostering a tumor microenvironment that is immunosuppressive and hostile. Therapeutic targeting miR130b with OX40 agoniztic antibody and lipid nanoparticles (LNP)-miR130b antagomir significantly inhibited Th17 cells and miR130b-overexpressing tumor growth in vitro and in vivo, and could be candidates for immunotherapeutic strategies for treating OX40-deficient B-cell lymphoma.^499^

### MiRNAs could also function as tumor suppressors in hematologic malignancies

MiRNAs can also function as tumor suppressors, and the prototypical examples are miR-15 and miR-16 in CLL.^475^ The deletion of 13q14 was the most prevalent genomic aberration detected in more than half of CLL cases.^500^ MiR15 and miR16 genes located within a 30-kb region of 13q14 that was lost in approximately 65% of cases of CLL. In 68 percent of CLL cases, allelic loss of this region was related with the deletion or downregulation of miR15 and miR16.^475^ This was the first time miRNA genes were found to be involved in cancer. The expression of miR-15 and miR-16 was then shown to be inversely associated to the expression of antiapoptotic Bcl-2, and both miR-15 and miR-16 targeted the 3' end of the BCL2 cDNA, thus adversely regulating BCL2 at the posttranscriptional stage. The loss of miR-15a and miR-16-1 caused BCL2 overexpression, which then worked as a driver of malignant transformation.^476^ Furthermore, miR-15 and miR-16 were also found to target the receptor tyrosine kinase-like orphan receptor 1 (ROR1) gene,^477^ which encodes an onco-embryonic antigen expressed on the surface of CLL cells.^501–503^ ROR1 is a receptor for Wnt5a,^504^ which could accelerate the development/progression of leukemia in a mouse model,^505^ and could be targeted by anti-ROR1 monoclonal antibodies for the treatment of CLL.^506,507^ Overall, miR-15/16 depletion enhanced the overexpression of ROR1 and BCL2, two important genes in the initiation and progression of CLL, acting as tumor suppressors in CLL.

### Some controversial miRNAs function as either oncogenes or tumor suppressors

Some miRNAs can function as either oncogenes or tumor suppressors, depending on the different contexts they encounter. The previously stated miR-17–92 cluster could be both oncogenic and tumor suppressive by targeting E2F1, which induces apoptosis in some cancers. c-MYC promoted the expression of the E2F1 gene,^507^ whereas E2F1 was able to induce c-MYC expression through positive feedback. miR-17-5p and miR-20a are capable of targeting E2F1, reducing the reciprocal activation of MYC/E2F1, and inhibiting c-MYC-mediated cell proliferation and carcinogenesis. MiR-29 works as a tumor suppressor by targeting the expression of TCL1, an oncogene in aggressive CLL,^508^ but transgenic mice that overexpressed miR-29 in B cells developed indolent CLL, suggesting that miR-29 plays an oncogenic role.^509^

### The physiologic role of long noncoding RNAs

LncRNAs are noncoding RNA transcripts longer than 200 nt that do not encode proteins.^510,511^ Pol II predominantly transcribes LncRNAs. Similar to mRNAs, they are frequently capped with 7-methyl guanosine (m7G) at their 5' ends, polyadenylated at their 3' ends, and posttranslationally spliced.^512^ LncRNAs are expressed at lower levels, are shorter, often lack an open reading frame (ORF), and are less evolutionarily conserved than protein-coding RNAs.^513–517^ Low expression of lncRNAs is associated with repressive histone modifications at their gene promoters,^518,519^ and they are also processed less efficiently than mRNAs.^518,520^ The majority of lncRNAs synthesized are retained in the nucleus.^514,516^ The remaining genes are spliced and transported to the cytoplasm.^521^ LncRNAs regulate gene expression at multiple levels, including epigenetic, transcriptional, and posttranscriptional levels.^512^ They exert biologic activity by interacting with DNA, RNA, and proteins through their functional modules which incorporate sequence motifs and secondary structures.^522^ They can act in cis or trans manners, and they can not only modulate their nearby chromatin state and gene expression but also leave their local transcription site and execute functions at distant cellular locations.^523^ LncRNAs can regulate chromatin by recruiting chromatin modifiers to the promoters of target genes, resulting in transcriptional repression or activation.^524–526^ Certain lncRNAs serve as decoys for chromatin modifiers by sequestering them from target gene promoters or interacting directly with chromatin.^527^ lncRNAs may also interact directly with DNA to produce RNA-DNA hybrids, such as triplexes^528,529^ or R-loops,^530–533^ which coordinate with chromatin modifiers or transcription factors to activate or repress the transcription of targeted genes.^530–532,534^ LncRNAs play a pivotal role in transcriptional regulation. By interacting with the transcription machinery, they modify recruited transcription factors or RNA polymeras,^535^ histone modification stations^535,536^ and chromatin accessibility^537,538^ to inhibit target gene expression. Some enhancer loci can be translated into lncRNAs, which are known as enhancer-associated lncRNAs (elncRNAs).^539,540^ ElncRNAs can promote target gene expression through the action of preexisting chromatin conformations^541^ or through the recruitment of chromatin-activating complexes to the promoters of target genes.^542^ They can recruit looping factors, thereby directly stimulating chromatin looping and distant gene expression.^543–545^ Multiple lncRNAs can form complex regulatory units to regulate the expression of target genes in concert.^546^ Specifically, regulatory DNA elements are just embedded in lncRNA loci and can stimulate the expression of neighboring genes.^547,548^ At the posttranscriptional level, lncRNAs also play important regulatory roles. They interact with proteins through their sequence motifs or structural motifs, subsequently interfering with mRNA splicing,^549,550^ regulating mRNA turnover,^551,552^ and modulating signaling pathways.^553^ In addition to binding with proteins, lncRNAs interact with RNAs through base pairing and then recruit proteins that regulate mRNA degradation.^554–556^ Furthermore, some lncRNAs are complementarily base paired with miRNAs and thus can competitively bind with miRNAs or “sponging” miRNAs, and modulate corresponding miRNA-targeted gene expression.^471,557^ Finally, lncRNAs are components of nuclear paraspeckle, and paraspeckles act as scaffolds for regulatory molecules.^558–561^

As described above, lncRNAs act extensively to regulate gene expression, and play important roles in development and various biological and disease contexts, including cancer. Accumulating evidence shows that lncRNAs are differentially expressed in tumors and are involved in malignant transformation and cancer progression.^456,562,563^ Similar to miRNAs, lncRNAs function as tumor suppressers and oncogenes or have dual effects.^482^ In hematologic malignancies, lncRNAs may influence cell proliferation, cell cycle regulation, and drug resistance, acting as tumor suppressors or oncogenes.

### LncRNA could function as an oncogene in hematologic malignancies

LUNAR1 (leukemia-induced noncoding activator RNA), a NOTCH-regulated lncRNA transcript in human T-ALL, is an example of a lncRNA functioning as an oncogene in hematologic malignancies.^564^ LUNRAR1 is controlled by the Notch1/Rpbj activator complex and has the ability to boost IGF1R mRNA expression and maintain IGF1 signaling, hence being essential for effective in vitro and in vivo T-ALL proliferation. LncRNA DANCR has been identified as a lncRNA related with LSCs in AML. DANCR is increased in functionally validated LSC-enriched populations and maintains the self-renewal and quiescence of LSCs. In vivo knockdown of Dancr in a primary murine model of AML slowed disease progression and increased mouse survival, confirming its oncogenic function in acute leukemia.^565^ TCLlnc1 was a newly identified lncRNA that acted as an oncogenic driver in the progression of PTCL.^566^ Serum TCLlnc1 level was correlated with tumor TCLlnc1 level. TCLlnc1 could be exploited as a possible biomarker for PTCL prognosis as it aws associated with high-risk clinical characteristics and poor prognosis.^566^ Overexpression of TCL1nc1 enhanced PTCL cell proliferation and migration in vitro and in vivo.^566^ TCLlnc1 interacted with the transcription activator heterogeneous nuclear ribonucleoprotein D (HNRNPD) and the Y-box binding protein-1 (YBX1) as a modular scaffold, thereby upregulating the transcription of the TGFB2 and TGFBR1 genes and activating the tumor growth factor-β signaling pathway, leading to lymphoma progression.^566^

### LncRNAs could also function as tumor suppressors in hematologic malignancies

LncRNAs may also function as tumor suppressors in hematologic malignancies, as illustrated by the downregulation of the lncRNA MEG3 in AML. MEG3 has been shown to inhibit leukemia cell growth via p53-dependent and p53-independent pathways, and WT1 and TET2 cooperate to upregulate MEG3 expression. MEG3 lowers cell proliferation, causes G0/G1 cell cycle arrest, and decreases AML leukemogenesis in animal models of AML.^567^ CML-specific LncRNA BGL3 is a critical regulator of cellular transformation mediated by Bcr-Abl. It was found that BGL3 overexpression makes leukemic cells more susceptible to apoptosis and suppresses Bcr-Abl-induced carcinogenesis. Transgenic animals expressing BGL3 were resistant to the Bcr-Abl-induced transformation of primary bone marrow, indicating that BGL3 is a tumor suppressor in CML.^568^

## Targeted therapy based on epigenetic regulation

### Targeting DNA methylation

#### DNA hypomethylating agents (HMAs)

**Fig. 3:**
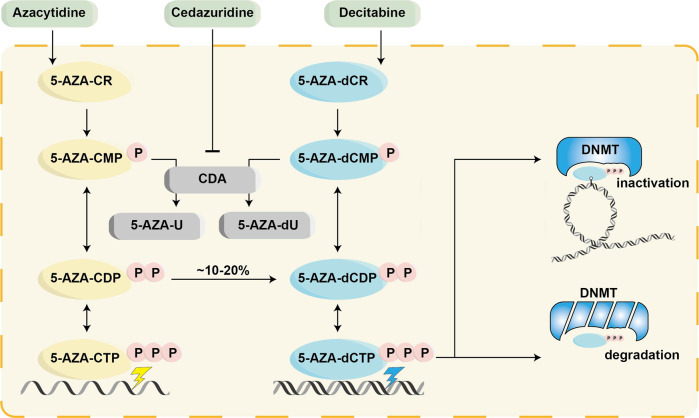
Intracellular metabolism of azacytidine and decitabine. Azacytidine (5-AZA-CR) and decitabine (5-AZA-dCR) are modified in a 3-step phosphorylation process by different metabolic pathways. 5-AZA-CTP/5-AZA-dCTP is incorporated into RNA or DNA by RNA/DNA polymerases, respectively. CDA can deaminate azacytidine and decitabine to inactive aza-uradine nucleosides. CDA cytidine deaminase, 5-AZA-CR 5-azacitidine, 5-AZA-CMP 5-azacitidine monophosphate, 5-AZA-CDP 5-azacitidine diphosphate, 5-AZA-CTP 5-azacitidine triphosphate, 5-AZA-U aza-uridine nucleosides, 5-AZA-dCR 5-aza-2’-deoxycytidine, 5-AZA-dCMP 5-aza-2’-deoxycytidine monophosphate, 5-AZA-dCDP 5-aza-2’-deoxycytidine diphosphate, 5-AZA-dCTP 5-aza-2’-deoxycytidine triphosphate, 5-AZA-dU deoxyuridine

Targeting abnormal DNA methylation has been explored, and DNA hypomethylating agents (HMAs) have been proposed. Two cytidine analogs, 5-azacytidine/vidaza (5-aza-CR, azacitidine) and 5-aza-2’-deoxycytidine/dacogen (5-aza-dCR, decitabine), have been approved to treat MDS by the FDA.^569,570^ The uridine-cytidine kinase phosphorylated azacitidine to a monophosphate derivative and then diphosphate and triphosphate forms. The triphosphate form (5-AZA-CTP) is primarily incorporated into RNA (∼80–90%) but also some into DNA (10–20%).^569,571,572^ Decitabine is a prodrug that undergoes a 3- step phosphorylation process intracellularly to be converted to the active moiety, decitabine triphosphate (5-AZA-dCTP), which is then incorporated into DNA by DNA polymerases. 5-AZA-dCTP forms a covalent complex with DNMTs, which leads to the trapping and degradation of the enzyme.^573,574^ It has been shown that cytidine deaminase (CDA) can deaminate azacytidine and decitabine to inactive aza-uradine nucleosides.^575^ Cedazuridine is a CDA inhibitor that prevents the degradation of decitabine when taken orally and increases the oral bioavailability of the drug. ASTX727 (decitabine/cedazuridine) has been approval by the USA and Canada for the treatment of MDS and CMML in July 2020.^576^ The details are shown in Fig. 3. Guadecitabine (SGI-110) is a second-generation HMA consists of a dinucleotide of decitabine and deoxyguanosine,^577^ which is an active metabolite of decitabine that prevents drug clearance by deamination by CDA has a longer half-life, bioavailability and greater safety.^578^ It is being tested in clinical trials for MDS and AML. OR-1200 and OR-2100, orally available single-compound prodrugs of decitabine, targeted aberrant DNA hypermethylation and repressed the development of CML and ATL.^579,580^ Myelosuppression was found to be the most common toxicity of azacitidine and decitabine, and adverse events (AEs) were generally transient, resolving during therapy.^581–583^ Zebularine is a DNMT-inhibiting cytosine nucleoside analog with stability in acidic environments and in aqueous solutions.^584^ Zebularine was shown to efficiently decrease AhR gene methylation in childhood ALL cells.^585^ However, zebularine required a near millimolar dose and had limited bioavailability in rodents (<7%) and primates (<1%); thus, it did not show good performance in a clinical trial.^586^ Although the TET family plays an essential role in DNA demethylation, TET protein inhibitors have yet to be tested for cancer treatment. The intracellular metabolism of azanucleosides is shown in Fig. 2. The completed clinical trials with results of targeting DNA methylation are concluded in Table 2.

**Table 2:** The completed clinical trials with results of targeting DNA methylation therapy

Conditions	Interventions	Phases	Patients	NCT number
Azacitidine				
AML/ALL/JMML/MDS	Azacitidine	2	17	NCT02458235
High-risk MDS	Azacitidine	2	72	NCT01599325
AL/MDS	Azacitidine+Sorafenib	1/2	60	NCT01254890
High-risk MDS/AML	Azacitidine+Sorafenib	2	16	NCT02196857
High-risk MDS/AML	Azacytidine+Lenalidomide	1/2	94	NCT01038635
R/R AML/AML with11q23 rearrangement	Azacitidine+Pinometostat	1/2	1	NCT03701295
CMML	Azacitidine	2	11	NCT01350947
High-risk MDS	Azacitidine	2	16	NCT00721214
AML	Azacitidine	2	24	NCT00387647
MDS/AML/MPN/CMML	Azacitidine/APR-246	1/2	55	NCT03072043
High-risk MDS	Azacitidine	NA	25	NCT00660400
Myelofibrosis	Azacitidine	2	34	NCT00569660
MDS/CMML	Azacitidine+Volasertib	1	5	NCT02201329
MDS	Azacitidine	3	40	NCT01186939
High-risk MDS/CMML/AML	Azacitidine+Pevonedistat	2	120	NCT02610777
MDS/AML/MM/NHL/HL	Azacitidine	1	31	NCT00652626
Low and intermediate-1 risk MDS	Azacitidine/Decitabine	2	113	NCT01720225
High-risk MDS	Azacitidine	4	44	NCT01201811
Advanced MDS	Azacytidine+Lenalidomide	1/2	37	NCT00352001
Relapsed AML	Azacitidine+Gemtuzumab ozogamicin	1/2	50	NCT00766116
Leukemia/AML/MDS	Azacitidine	3	187	NCT00887068
MM	Azacitidine	NA	17	NCT01050790
MDS	Azacitidine	2	25	NCT00384956
AML	Azacitidine/Conventional Care Regimen	3	488	NCT01074047
Poor-risk AML/MDS	Azacitidine+Sargramostim	2	25	NCT01700673
AML	Azacitidine/Lenalidomide/Best Supportive Care	2	88	NCT01358734
Leukemia	Azacytidine/PKC412	1/2	54	NCT01202877
AML	Azacitidine+Lenalidomide	1/2	45	NCT00890929
AML/MDS	Azacitidine+Vorinostat	2	110	NCT00948064
AML	Azacitidine+MLN4924	1	64	NCT01814826
AML/MDS/CMML	Azacitidine	2	43	NCT01083706
R/R AML	Azacitidine+Lenalidomide	2	37	NCT01743859
MDS/AML/CMML	Azacitidine+PF-04449913 (Glasdegib)	1	73	NCT02367456
AML/MDS	Azacytidine	2	40	NCT02497404
AML	Azacitidine+Lenalidomide	1/2	31	NCT01016600
MDS/CMML/AML	Azacytidine+Panobinostat (LBH589)	1/2	113	NCT00946647
MDS/AML	Azacytidine+Valproic Acid+ATRA	2	34	NCT00326170
Refractory MM	Azacitidine Lenalidomide+Dexamethasone	1/2	45	NCT01155583
MDS	Azacytidine	2	151	NCT00102687
MDS	Azacytidine+Etanercept	1/2	32	NCT00118287
AML/CMML/MDS	Azacitidine/+Entinostat	2	197	NCT00313586
AML	Azacitidine+Midostaurin	1/2	34	NCT01093573
R/R AML/high-risk MDS	Azacytidine+Ara-C	1/2	36	NCT00569010
AML/MDS	Azacytidine+Valproic Acid+Ara-C	2	11	NCT00382590
CML	Azacitidine	2	24	NCT00813124
Childhood R/R ALL/AML	Azacytidine+Chemotherapy	1	15	NCT01861002
High-risk MDS	Azacitidine/Conventional Care	3	358	NCT00071799
R/R DLBCL	Azacitidine Vorinostat	1/2	17	NCT01120834
High-risk MDS	Azacytidine+Valproic Acid+ATRA	2	62	NCT00439673
R/R AML	Azacitidine+Vorinostat+Gemtuzumab Ozogamicin	1/2	52	NCT00895934
DLBCL	Azacytidine+R-CHOP	1/2	14	NCT01004991
Refractory lymphoma	Azacytidine -SAHA-GBM	1/2	61	NCT01983969
AML	Intensive Chemotherapy/+Glasdegib/+Azacitidine/+Glasdegib	3	730	NCT03416179
AML	Azacitadine+Pracinostat	2	50	NCT01912274
AML/MDS	Azacitidine (CC-486)	1/2	31	NCT01835587
MDS	Eltrombopag/+Hypomethylating Agent	2	29	NCT01893372
Decitabine				
Relapsed AML	Decitabine+Pembrolizumab	1/2	10	NCT02996474
AML/high-risk MDS	Decitabine+Vosaroxin	1/2	66	NCT01893320
AML/MDS	Decitabine	2	114	NCT01687400
AML	Decitabine	2	74	NCT01786343
AML	Decitabine+Talacotuzumab (JNJ-56022473; anti CD123) v.s. Decitabine	2/3	326	NCT02472145
MDS	Decitabine	2	37	NCT00744757
AML/MDS	Decitabine	2	10	NCT00760084
MDS/AML	Decitabine/+Valproic Acid	2	153	NCT00414310
Low and intermediate-1 risk MDS	Decitabine v.s. Azacitidine	2	113	NCT01720225
High-risk MDS	Decitabine+Clofarabine	2	42	NCT00903760
AML/high-risk MDS	Decitabine+Gemtuzumab Ozogamicin	2	43	NCT00882102
MDS	Decitabine+Arsenic Trioxide+Ascorbic Acid	2	7	NCT00621023
AML/high-risk MDS	Decitabine+Gemtuzumab Ozogamicin	2	71	NCT00968071
R/R AML/high-risk MDS	Decitabine+Mitoxantrone Hydrochloride+Etoposide+Cytarabine	1/2	52	NCT01729845
Low or intermediate-1 risk MDS	Decitabine	2	67	NCT00619099
MDS	Decitabine	3	135	NCT01751867
AML/MDS	Decitabine	1	16	NCT01378416
AML	Decitabine	2/3	50	NCT00398983
AML/high-risk MDS/MPN	Decitabine+Cytarabine	NA	12	NCT02121418
AML/MDS	Decitabine+Vorinostat	1	71	NCT00479232
AML/high-risk MDS	Decitabine+GCLAM	1/2	28	NCT02921061
MDS	Decitabine	1	39	NCT00796003
MDS/AML	Decitabine+LBH589	1/2	52	NCT00691938
Advanced MDS	Decitabine	2	99	NCT00260065
AL	Decitabine+Clofarabine+Idarubicin+Cytarabine	1/2	65	NCT01794702
AML	Decitabine	3	485	NCT00260832
AML/high-risk MDS	Decitabine+Clofarabine+Cytarabine	2	122	NCT00778375
MDS	Decitabine	2	128	NCT00067808
AML	Decitabine+Cytarabine	2	44	NCT01829503
AML	Decitabine	2	55	NCT00358644
High-risk MDS	Decitabine+Vorinostat+Natural killer (NK) cells	2	9	NCT01593670
AML	Decitabine	2	546	NCT00416598
AML	Decitabine	2	55	NCT00492401
R/R AML	Decitabine+total body irradiation	2	20	NCT01707004
MDS	Decitabine	1/2	25	NCT01165996
AML/MDS	PF-04449913+Ara-C+Decitabine+Daunorubicin+Cytarabine	2	255	NCT01546038
Guadecitabine				
MDS	SGI-110 (guadecitabine)	2	22	NCT03075826
AML	SGI-110 (guadecitabine)	3	815	NCT02348489
AML	SGI-110 (guadecitabine)/+Idarubicin or Cladribine	2	44	NCT02096055
AML	SGI-110 (guadecitabine)	1	21	NCT02293993
ASTX727 (Cedazuridine/Decitabine)				
MDS/CMML	Decitabine/Cedazuridine	1/2	130	NCT02103478

*AL* acute leukemia, *ALL* acute lymphoblastic leukemia, *AML* acute myeloid leukemia, *Ara-C* cytosine arabinoside, *ATRA* all-trans retinoic acid, *CMML* chronic myelomonocytic leukemia, *DLBCL* diffuse large B-cell lymphoma, *HL* Hodgkin lymphoma, *JMML* juvenile myelomonocytic leukemia, *MDS* myelodysplastic syndrome, *MM* multiple myeloma, *MPN* myeloproliferative neoplasm, *NHL* non-Hodgkin lymphoma, *R/R* relapsed/refractory

Furthermore, many non-nucleoside analogs have been reported as DNA HMAs, which are usually small-molecule inhibitors and directly target catalytic sites rather than being incorporated into DNA. GSK3685032 was a DNMT1-selective inhibitor that could inhibit DNA methylation, transcriptional activation and cancer cell proliferation inhibition in vitro.^587^ Epigallocatechin gallate (EGCG) was the most effective polyphenol in green tea and inhibited the proliferation of APL, AML, and CML cells by inhibiting the expression of DNMTs.^588–592^ EGCG also induced the demethylation and transcription of the p16 gene in the MM cell line CA46.^593^ Thymoquinone (TQ) was a major component of *Nigella sativa* seeds, inducing DNMT1 dysfunction,^594^ and could enhance demethylation in AML.^595^ TQ could also mediate apoptosis by targeting UHRF1 in Jurkat cells.^212^ The combination of DFMO and TQ decreased the expression of the *UHRF1*, *DNMT1* and *HDAC1* genes in Jurkat cells.^211^ Quercetin, an important dietary flavonoid, eliminated DNMT1 and DNMT3a expression and enhanced the apoptosis of leukemia cells.^596^ Harmine, a beta-carboline alkaloid derivative of Peganum harmala, could inhibit DNMT1 expression in leukemia cells.^597^ Curcumin, a component of the popular Indian spice turmeric, could decrease DNMT1 expression and played an anti-leukemic role in AML.^598^ Novel curcumin liposomes modified with hyaluronan was found to downregulate DNMT1 expression and played an anti-leukemic role in AML.^599^ NT1721, a novel epidithiodiketopiperazine, was shown to deplete DNMT1 protein levels, causing the re-activation of silenced tumor suppressor genes.^600^ Furthermore, parthenolide, a major component of the feverfew medicinal plant, reduced the expression of DNMT1 in primary effusion lymphoma models.^601^ Oridonin was an ent-kaurene diterpenoid extracted from the Chinese herb Rabdosia rubescens, and was shown to inhibit *DNMT3A*^R882^ mutation-driven clonal hematopoiesis and leukemia.^602^ RG108, a DNA methyltransferase inhibitor, could inhibit the activity of DNMT enzymes and cause demethylation.^603^ Emodin, an active component in the roots and rhizomes of numerous Chinese medicinal herbs, was demonstrated to inhibit human lymphoma Raji cell proliferation by UHRF1-DNMT3A-∆Np73 pathways.^604^ Berberine was a main constituent of optidis rhizome and an isoquinoline alkaloid, and it was reported to repress the expression of DNMT1 and DNMT3B, and to induce apoptosis in the human MM cell line U266 through p53 promoter hypomethylation.^605^ MG98, oligonucleotide antisense to DNMT1, displayed no pharmacodynamic or clinical activity when administered to patients with high-risk MDS.^606^

#### Combination treatment of HMAs with immune treatment

HMAs in combination with immune checkpoint inhibitors were widely used.^607^ CD47 was a macrophage checkpoint and can be targeted for AML and MDS,^608^ and its inhibitor in combination with azacitidine had increased efficacy of AML and higher-risk MDS, especially in patients with *TP53* mutation.^609^ T-cell immunoglobulin domain and mucin domain-3 (TIM-3) was a T-cell immune checkpoint, and highly expressed in LSCs. Sabatolimab (an anti-TIM-3 monoclonal antibody) in combination with HMAs had durable responses in HR-MDS and AML patients.^607^ In a phase 1b trial, ipilimumab (CTLA-4) showed limited efficacy in HR-MDS patients after failing from HMAs.^610^ HMAs could increase the expression level of PD-1, PD-L1, PD-L2, and CTLA-4 in patients with AML and MDS, which caused HMA resistance.^610^ Therefore, there are clinical trials combining PD-1/PD-L1 inhibitors with HMAs in AML and MDS patients. The ORR rate and median OS were higher in azacitidine plus nivolumab plus ipilimumab than azacitidine plus nivolumab group.^611^ While, high-risk MDS patients after the failure of HMAs did not benefit from pembrolizumab.^612^ Decitabine in combinatipn with PD-1 inhibitors was also used in classical HL (cHL) treatment. Decitabine increased the efficacy of anti-PD-1 antibody in refractory cHL, even in patients with anti-PD-1 monotherapy resistance.^613,614^ The completed clinical trials with HMAs plus other treatments, including chemotherapy, PD-1 inhibitor, are listed in Table 2.

#### IDH inhibitors

The IDH1 inhibitors ivosidenib, olutasidenib, and IDH2 inhibitor enasidenib have been approved by FDA for patients with relapsed/refractory *IDH1* mutant AML. In a phase I study, the ORR of ivosidenib was 41.6%, the CR and CRi was 30.4%.^615^ In a phase 1 clinical trial, the ORR of olutasidenib was 41% and 46% in combination with azacitidine in relapsed/refractory AML.^616^ The ORR of enasidenib in relapsed/refractory AML patients with *IDH2* mutations was 40.3%, and the CR was 19.3% in a phase I/II study.^617^ The median OS for all pateints was 9.3 months, but 19.7 months for patients who achieved CR.

Furthermore, the IDH1 inhibitor ivosidenib combined with azacytoside as a first-line treatment for AML patients carrying an *IDH1* mutation has been approved by the FDA. In a phase Ib clinical trial, the ORR was 78.3% and the CR was 60.9%.^618^ In a phase 3 study, the EFS was longer in the combination group than in the placebo-and-azacitidine group (HR = 0.33, *P* = 0.002). The median OS in ivosidenib and azacitidine group (24.0 months) was longer than the placebo and azacitidine group (7.9 months) (HR = 0.44, *P* = 0.001).^619^

The ongoing clinical trials are listed in Table 3. The clinical trials mainly focus on AML, and MDS. The IDH inhibitor combined with other treatments, including chemotherapys, PD-1 inhibitor, MEK inhibitor, and FLT3/AXL inhibitors, are listed in Supplementary Table 1.

**Table 3:** The clinical trials of IDH inhibitors

NCT number	Conditions	Interventions	Phase	Number enrolled
NCT04176393	R/R AML	Ivosidenib	Phase 1	30
NCT03564821	IDH1-mutated myeloid neoplasms	Ivosidenib	Phase 1	18
NCT03245424	AML	Ivosidenib	NA	NA
NCT02074839	R/R AML/MDS/other IDH1-mutated positive hematologic malignancies	Ivosidenib	Phase 1	291
NCT03839771	AML/MDS	Ivosidenib	Phase 3	968
NCT03503409	AML/MDS	Ivosidenib	Phase 2	68
NCT05282459	AML	Enasidenib	Phase 1/2	48
NCT04203316	R/R AML	Enasidenib	Phase 2	10
NCT03515512	AML/CML	Enasidenib	Phase 1	23
NCT01915498	Hematologic neoplasms	Enasidenib	Phase 1	345
NCT03720366	AML	Enasidenib	Phase 1	40
NCT03728335	AML	Enasidenib	Phase 1	15
NCT03881735	R/R AML	Enasidenib	Phase 2	0
NCT03744390	AML/MDS	Enasidenib	Phase 2	68
NCT03723057	AML	Enasidenib	NA	NA
NCT04522895	AML/MDS/CML	Enasidenib	Phase 2	50

*AML* acute myeloid leukemia, *CML* chronic myeloid leukemia, *MDS* myelodysplastic syndrome, *NA* not applicable, *R/R* relapsed/refractory

### Targeting HDAC

**Fig. 4:**
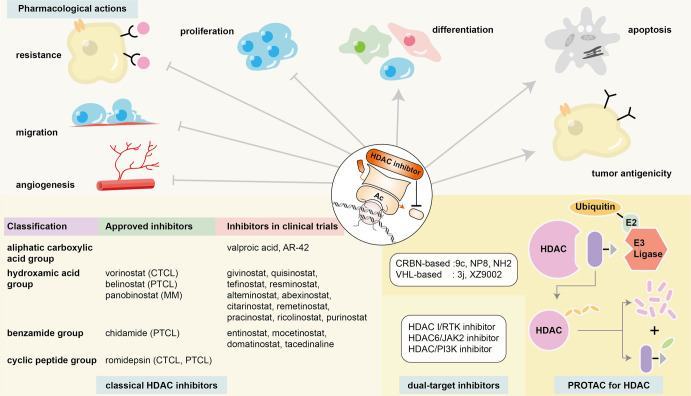
Classic HDAC inhibitors, dual-target HDAC inhibitors, and RPOTAC for HDAC. HDAC inhibitors not only inhibit angiogenesis, cell migration, resistance to treatment, and cell proliferation but also stimulate cell differentiation, apoptosis, and tumor antigenicity. The development of HDAC-targeting treatment ranges from classic HDAC inhibitors, dual-target HDAC inhibitors, and PROTAC for HDACs. Classic HDAC inhibitors are divided into four groups based on structures. The first group is aliphatic carboxylic acid group. None of the HDAC inhibitors of this group have been approved for hematologic malignancy treatment. The second group is hydroxamic acid group and includes vorinostat, belinostat, and panobinostat, which has been approved for treatment of CTCL, PTCL, and MM, respectively. The third group is benzamide group and includes chidamide, which has been approved for the treatment of PTCL. The last group is the cyclic peptide group and includes romidepsin, which has been approved for the treatment of CTCL and PTCL. Furthermore, dual-target HDAC inhibitors have been explored in hematologic malignancy treatment, which also target RTK, JAK2, and PI3K. In additions, PROTAC for HDACs has been developed recently

HDAC inhibitors not only trigger cell differentiation, apoptosis, autophagy, and cell cycle arrest but also modulate immune reactions and inhibit angiogenesis in various hematologic malignancies and some solid tumors.^620^ It has been hypothesized that malignant cells are more vulnerable to epigenetic treatment, rendering the specificity and selectivity of malignant cells over normal cells.^351^ A number of HDAC inhibitors have been developed and demonstrated effective in treating hematologic malignancies. The U.S FDA and China National Medical Products Administration (NMPA) have approved five HDAC inhibitors for the treatment of T-cell lymphoma, MM, and breast cancer. The classical inhibitors, dual-target inhibitors, and PROTAC for HDAC are discussed below (Fig. 4).

#### Classic HDAC inhibitors

Classic HDAC inhibitors can be divided into four groups based on their zinc-binding group: the aliphatic carboxylic acid group, hydroxamic acid group, benzamide group, and cyclic peptide group. In general, the aliphatic carboxylic acid group displays weak deacetylation ability and mild selectivity for class I HDACs. The hydroxamic acid group displays strong pan-deacetylation ability with an IC_50_ value of nM level. The benzamide group and cyclic peptide group have strong selectivity for class I HDAC.

In the aliphatic carboxylic acid group, which inhibits class I HDACs, sodium butyrate has been investigated in the chemoprevention of breast cancer and colon cancer. Valproic acid has been approved for seizure treatment, and several clinical trials are investigating its combination with chemotherapy to treat CLL, AML, MDS, and lymphoma. AR-42 resembles phenylbutyrate in structure and inhibits HDACs with an IC_50_ of 30 nM. In AML, AR-42 was shown to repress the NF-κB pathway, downregulate oncogenic Kit through HSP90 disturbance, and specifically trigger the apoptosis of LSCs without affecting normal HSCs.^621^ In MM, AR-42 could trigger cell cycle arrest and the apoptosis of MM cells, and downregulated CD44, which represents resistance to immunomodulating agents (IMiDs) and dexamethasone.^622,623^ However, in clinical trials, AR-42 demonstrated only mild disease control of MM and a relatively low overall response rate (ORR) (23.1%) when combined with decitabine in AML, impeding its further application in hematologic malignancy treatment.^624,625^

In the hydroxamic acid group, vorinostat, belinostat, and panobinostat have been approved by the FDA, while ricolinostat and citarinostat are being investigated in clinical trials. Vorinostat, also named SAHA, inhibits class I, II, and IV HDACs, and in 2006 was the first HDAC inhibitor approved by the U.S. FDA to treat cutaneous T-cell lymphoma/leukemia (CTCL). For CTCL patients who had previously received two lines of systemic treatment, the ORR of vorinostat treatment was 30%, and the median time to progression was 202 days. Serious adverse events (sAEs) of grade 3 or above included pulmonary embolism (5%) and fatigue (4%).^626,627^ Combination treatment of vorinostat with rituximab, proteasome inhibitors (PIs), and HMA is under investigation in lymphoma. In MM, the combination of vorinostat, PIs and IMiDs is being explored in clinical trials. Furthermore, various clinical trials exploring vorinostat combined with chemotherapy to treat AML, MDS, ALL, CML, and CLL and to prevent graft-versus-host disease (GVHD) have been completed or are ongoing (Table 4). In 2014, belinostat, also known as PXD101, which inhibits class I and II HDACs with nanomolar IC_50_ values, was the third HDAC inhibitor approved by the U.S. FDA to treat relapsed/refractory (RR) PTCL. For R/R PTCL patients, the ORR of belinostat was 25.8%, and the median duration of response (DoR) was 8.4 months. The sAEs of grade 3 or above were hematologic AEs (6.2–10.9%), pneumonia (5.4%), dyspnea (6.2%), and fatigue (5.4%).^628^ In addition to PTCL, belinostat has been investigated alone or in combination with rituximab, idarubicin, HMA, NEDD8 activating enzyme inhibitors, and PIs in the treatment of R/R aggressive B-cell lymphoma, AML, MDS, and MM in various clinical trials (Table 4). In 2015, panobinostat (also named LBH589), which inhibits class I, II, and IV HDACs, was the fourth HDAC inhibitor approved by the U.S. FDA to treat MM. In the PANORAMA1 trial, R/R MM patients received bortezomib and dexamethasone combined with panobinostat or a placebo. The panobinostat group displayed significantly longer PFS (median 11.99 months) and higher CR or near CR rate (27.6%) than the placebo group, although no significant benefit in OS was discovered.^629,630^ In the PANORAMA2 trial, heavily pretreated bortezomib-refractory MM patients received treatment with panobinostat, bortezomib, and dexamethasone, and the ORR was 34.5%.^631^ In the MUK-six trial, R/R MM patients received treatment with panobinostat, bortezomib, thalidomide and dexamethasone, and the ORR was 91%.^632^ However, the U.S. FDA has issued a warning regarding an increased risk of diarrhea, cardiac toxicity, and abnormal electrocardiogram manifestations with the use of panobinostat. Various clinical trials have examined panobinostat alone or combined with PIs, IMiDs, and everolimus in MM and lymphoma. In addition, AML, MDS, myelofibrosis (MF), and GVHD were also explored to confirm the safety and efficacy of panobinostat combined with corresponding standard therapy (Table 4). Apart from the approved HDAC inhibitors, various not-yet-approved inhibitors have been explored in clinical trials. Givinostat, also named ITF2357, inhibits HDAC1 and HDAC3 and has been demonstrated to regulate NFE2 and C-MYB hematopoietic transcription factors and trigger the apoptosis in *JAK2*^V617F^-positive MPN.^633,634^ It was also shown to trigger apoptosis, regulate the cell cycle, and promote differentiation in T-ALL and B-ALL.^635–637^ Several clinical trials investigated its efficacy in MPN and HL, and the ORR of givinostat in the treatment of polycythemia vera was more than 80%, accompanied by a favorable safety profile.^638–640^ Quisinostat, also named JNJ-26481585, inhibits HDAC1, HDAC2, and HDAC3 with nanomolar IC_50_ value. In MM cells, quisinostat stimulated the caspase cascade and upregulated p21, which in turn triggered apoptosis and cell cycle arrest in vitro. Quisinostat alone could also inhibit angiogenesis and significantly reduce the tumor burden in MM mouse models.^641,642^ Quisinostat combined with bortezomib significantly remodeled bone structure and reduced bone disease.^643^ When combined with bortezomib and dexamethasone, quisinostat exerted an ORR of 88.2% in R/R MM patients.^644^ The cutaneous response rate to quisinostat in previously treated CTCL patients was 24%.^645^ Tefinostat, also named CHR-2845, is a pan-HDAC inhibitor that exerts anti-leukemic activity in monocytoid-lineage leukemia in vitro.^646^ In a phase I trial including 18 patients with R/R hematologic malignancies, tefinostat displayed a good tolerance profile and showed early signs of response in a CMML patient and an AML-M2 patient.^647^ Resminostat, also named 4SC-201, inhibits HDAC1, HDAC3, and HDAC6. Resminostat repressed proliferation and induced the apoptosis and G0/G1 cell cycle arrest of MM cells in vitro. Resminostat also disturbed the Akt signaling pathway by reducing 4E-BP1 and p70S6k phosphorylation.^648^ In addition, resminostat exerted an antitumor effect when combined with ruxolitinib in CTCL models.^649^ In the phase II SAPHIRE trial, resminostat showed an ORR of 34% in R/R HL patients.^650^ Abexinostat, also named PCI-24781, is a pan-HDAC inhibitor that mainly targets HDAC1. Abexinostat can stimulate apoptosis via the NFκB pathway and remodel chromatin in lymphoma cells.^651,652^ Various clinical trials have examined abexinostat in FL, MCL, and DLBCL. The ORR of abexinostat in R/R FL patients was 64.3%, and that in R/R MCL patients was 27.3%.^653^ Alteminostat, also named CKD-581, is a pan-HDAC inhibitor that inhibits proliferation; downregulates c-Myc, BCL-2, BCL-6, and MCL-1; and upregulates p53, p21, and H2AX phosphorylation in MM cells and double-hit/double-expressor DLBCL cells.^654,655^ In a phase I trial including MM and lymphoma patients who were refractory to standard treatment, alteminostat exhibited a favorable safety profile with a relatively low PR rate of 5.6%.^656^ Citarinostat, also named ACY-241, inhibits HDAC6 and HDAC3 and exerts synergistic antimyeloma effects combined with pomalidomide in vitro and in MM mouse models.^657^ Several clinical trials have explored its safety and efficacy in MM patients. Remetinostat, also named SHP-141, inhibits HDAC1, HDAC3, and HDAC6 and was designed to exert efficacy specifically and locally in the skin.^658^ Clinical trials have examined remetinostat in early stage CTCL. Pracinostat, also named SB939, is a pan-HDAC inhibitor that showed synergism with JAK2 inhibitors in AML models and increased sensitivity to BCR-ABL kinase inhibitors in CML.^659,660^ Several clinical trials have investigated its efficacy in MDS and MF. Pracinostat showed only modest or mild efficacy in MF and showed an even lower ORR when combined with HMAs compared with HMA alone, preventing its further application to the two diseases.^661–664^ Ricolinostat, also named ACY-1215, is a first-in-class HDAC6 inhibitor. Ricolinostat combined with bortezomib caused sustained endoplasmic reticulum stress and apoptosis by stimulating caspase-3, caspase8, caspase 9, and poly (ADP) ribosome polymerase in MM cells in vitro. In a plasmacytoma MM model and disseminated MM model, ricolinostat combined with bortezomib significantly inhibited tumor growth and extended OS.^665^ It also increased sensitivity to daratumumab by upregulating CD38 expression in MM cells.^666^ A similar antitumor effect was also found in lymphoma.^667^ A combination treatment of ricolinostat with an immune checkpoint inhibitor and ibrutinib was effective in CLL and FL, respectively, in vitro and in vivo.^668,669^ Several clinical trials are examining its effect in R/R MM, CLL, and lymphoma. For R/R MM patients, ricolinostat combined with bortezomib and dexamethasone showed an ORR of 37%.^670^ and ricolinostat combined with lenalidomide and dexamethasone displayed an ORR of 55%.^671^ Purinostat, an HDAC I/IIb inhibitor, has demonstrated promising antitumor effects in B-ALL and DLBCL mouse models and is being explored in B-cell lymphoma and MM in China.^672,673^

**Table 4:** The clinical trials of approved HDAC inhibitors

Conditions	Interventions	Phase	*N*	NCT number	Status
Vorinostat					
CTCL	Vorinostat	1	10	NCT00771472	Completed
Advanced CTCL	Vorinostat	2	74	NCT00091559	Completed
ND T-cell lymphoma	Vorinostat+CHOP	1/2	14	NCT00787527	Completed
ND T-cell lymphoma, MCL, R/R other types of lymphoma	Vorinostat+Rituximab+Ifosfamide+Carboplatin+Etoposide	1/2	29	NCT00601718	Completed
ENKT	Vorinostat+Azacitidine	1	18	NCT00336063	Active, not recruiting
Low-grade NHL	Vorinostat	2	37	NCT00253630	Completed
Indolent NHL	Vorinostat+Rituximab	2	30	NCT00720876	Completed
R/R NHL, AML, ALL, CML	Vorinostat+Decitabine	1	80	NCT00275080	Completed
Relapsed NHL	Vorinostat+Combination Chemotherapy	2	30	NCT04220008	Not yet recruiting
Indolent B-cell NHL, MCL	Vorinostat	2	56	NCT00875056	Completed
R/R MCL, DLBCL	Vorinostat+Bortezomib	2	65	NCT00703664	Completed
MCL, R/R B-NHL, R/R CLL	Vorinostat+Cladribine+Rituximab	2	57	NCT00764517	Completed
ND stage II-IV DLBCL	Vorinostat+Rituximab+Combination Chemotherapy	1/2	83	NCT00972478	Active, not recruiting
R/R DLBCL, FL, HL	Vorinostat+Pembrolizumab	1	52	NCT03150329	Completed
R/R DLBCL	Vorinostat+Azacitidine	1/2	17	NCT01120834	Completed
Relapsed DLBCL	Vorinostat	2	18	NCT00097929	Completed
Elderly relapsed DLBCL	Vorinostat+Cyclophosphamide, Etoposide, Prednisone and Rituximab	1/2	30	NCT00667615	Completed
R/R B-cell lymphoma	Vorinostat+Carfilzomib	1	20	NCT01276717	Completed
R/R advanced HL	Vorinostat	2	27	NCT00132028	Completed
HIV-related DLBCL, other aggressive B-cell lymphoma	Vorinostat+Rituximab+Combination Chemotherapy	1/2	107	NCT01193842	Completed
Lymphoma	Vorinostat+Niacinamide+Etoposide	1	40	NCT00691210	Completed
High-risk lymphoma	Vorinostat after stem cell transplantation	1	23	NCT00561418	Completed
Relapsed lymphoma	Vorinostat	1	10	NCT00127140	Completed
R/R lymphoma	Vorinostat+Gemcitabine+Busulfan+ Melphalan+Stem Cell Transplant	1	78	NCT01421173	Completed
R/R lymphoma	Vorinostat+Alisertib	1	34	NCT01567709	Completed
NHL after ASCT	Vorinostat+Bortezomib	2	27	NCT00992446	Completed
ND MM	Vorinostat+Bortezomib+Lenalidomide+Dexamethasone	1	30	NCT01038388	Completed
ND MM in maintenance treatment	Vorinostat+Lenalinomide	3	4420	NCT01554852	Active, not recruiting
R-resistant MM	Vorinostat+Lenalinomide+Dexamethasone	1/2	25	NCT01502085	Completed
R/R MM	Vorinostat+Bortezomib	1	34	NCT00111813	Completed
R/R MM	Vorinostat+Bortezomib	1	9	NCT00858234	Completed
R/R MM	Vorinostat+Bortezomib	1	40	NCT00310024	Completed
R/R MM	Vorinostat+Bortezomib	2	143	NCT00773838	Completed
R/R MM	Vorinostat+Lenalinomide+Dexamethasone	1	31	NCT00642954	Completed
R/R MM	Vorinostat+Bortezomib+Doxorubicin+Dexamethasone	1/2	34	NCT01394354	Completed
R/R MM	Vorinostat+Carfilzomib+Lenalidomide+Dexamethasone	1/2	17	NCT01297764	Active, not recruiting
MM after ASCT	Vorinostat+Lenalinomide	1	19	NCT00729118	Completed
MM after ASCT	Vorinostat+Bortezomib	2	31	NCT00839956	Completed
MM	Vorinostat+Bortezomib	3	637	NCT00773747	Completed
MM	Vorinostat+Bortezomib+ Dexamethasone	2	16	NCT01720875	Completed
Young ND AML	Vorinostat/+Idarubicin and Cytarabine v.s. Cytarabine+Daunorubicin Hydrochloride	3	754	NCT01802333	Completed
ND AML, MDS	Vorinostat+Azacitidine	2	110	NCT00948064	Completed
R/R AML	Vorinostat+Temozolomide	2	23	NCT01550224	Completed
R/R AML, MDS	Vorinostat+Decitabine+Cytarabine	1	17	NCT01130506	Completed
R/R AML, ALL, CML	Vorinostat+Flavopiridol	1	24	NCT00278330	Completed
R/R AML	Vorinostat+Decitabine+Fludarabine+Cytarabine+Filgrastim (G-CSF)	1	37	NCT03263936	Completed
R/R or poor prognosis AML, MDS, CML, ALL	Vorinostat+Decitabine	1	50	NCT00357708	Completed
R/R AML	Vorinostat+venetocla+azacitidine+Cytarabine+Fludarabine+Filgrastim	1	40	NCT05317403	Not yet recruiting
Elder R/R AML	Vorinostat+Azacitidine+Gemtuzumab Ozogamicin	1/2	52	NCT00895934	Completed
Poor-risk AML, high-risk MDS	Vorinostat+Sorafenib	1	15	NCT00875745	Completed
Pediatric or young adult AML, MDS	Vorinostat+Azacytidine after alloSCT	1	15	NCT03843528	Recruiting
Complex/poor-risk cytogenetics AML or FLT3-ITD	Vorinostat+Sorafenib+Bortezomib	1/2	37	NCT01534260	Completed
AML	Vorinostat	2	37	NCT00305773	Completed
AML, MDS	Vorinostat+Decitabine	1	71	NCT00479232	Completed
AML, MDS	Vorinostat+Azacitidine	1/2	135	NCT00392353	Active, not recruiting
AML, high-risk MDS	Vorinostat/+Azacitidine	2	260	NCT01617226	Completed
AML, high-risk MDS	Vorinostat+Idarubicin+Cytarabine	2	106	NCT00656617	Completed
R/R leukemia, MDS	Vorinostat+Idarubicin	1	40	NCT00331513	Completed
AML before alloSCT	Vorinostat+Fludarabine Phosphate+Clofarabine+Busulfan	1	70	NCT02083250	Completed
High-risk MDS	Vorinostat+Low Dose Cytarabine	1/2	52	NCT00776503	Completed
High-risk MDS before CD3-/CD19- NK Cells Infusion	Vorinostat+Decitabine	2	9	NCT01593670	Completed
High-risk MDS, CMML	Vorinostat/Lenalidomide/- +Zzacitidine	2	282	NCT01522976	Completed
Acute leukemia, MDS, MPN	Vorinostat+Cytarabine+Etoposide	1	25	NCT00357305	Completed
Infant ALL	Vorinostat+Bortezomib+Chemotherapy	1/2	50	NCT02553460	Active, not recruiting
Accelerated phase or blastic phase CML, ALL	Vorinostat+Dasatinib	1	5	NCT00816283	Completed
ND CLL/SLL	Vorinostat+Fludarabine+Cyclophosphamide+Rituximab	1/2	40	NCT00918723	Completed
Reduced intensity, related donor stem cell transplant	Vorinostat+Tacrolimus+Mycophenolate	1/2	61	NCT00810602	Completed
Unrelated stem cell transplant	Vorinostat+Tacrolimus+Methotrexate	2	26	NCT01790568	Completed
AlloSCT	Vorinostat+Tacrolimus+Methotrexate+Mycophenolate Mofetil+Cyclophosphamide	1/2	49	NCT03842696	Recruiting
Stem cell transplant	Vorinostat+Tacrolimus+Methotrexate	2	12	NCT01789255	Completed
Belinostat					
PTCL	Belinostat+CHOP	1	23	NCT01839097	Completed
R/R PTCL	Belinostat	2	129	NCT00865969	Completed
R/R BL, DLBCL	Belinostat	2	22	NCT00303953	Completed
R/R NHL	Belinostat+Carfilzomib	1	19	NCT02142530	Completed
Relapsed aggressive high-risk lymphoma	Belinostat+Rituximab+Yttrium Y 90 Ibritumomab Tiuxetan	2	5	NCT01686165	Completed
Adult T-cell leukemia/lymphoma	Belinostat+Zidovudine	2	20	NCT02737046	Recruiting
R/R AML	Belinostat+Pevonedistat	1	30	NCT03772925	Recruiting
AML unsuitable for standard intensive therapy	Belinostat+Idarubicin	1/2	41	NCT00878722	Completed
AML	Belinostat	2	12	NCT00357032	Completed
R/R acute leukemia, MDS	Belinostat+Bortezomib	1	41	NCT01075425	Completed
AML, ALL, CML, MDS	Belinostat+Azacitidine	1	56	NCT00351975	Completed
MDS	Belinostat	2	21	NCT00357162	Completed
Advanced MM	Belinostat+Dexamethsone	2	25	NCT00131261	Completed
Panobinostat					
R/R MM	Panobinostat+Bortezomib+Dexamethasone	3	767	NCT01023308	Completed
R/R MM	Panobinostat+Bortezomib+Dexamethasone	2	249	NCT02654990	Active, not recruiting
R/R MM	Panobinostat+Bortezomib+Dexamethasone	2	31	NCT02290431	Completed
Relapsed and bortezomib-refractory MM	Panobinostat+Bortezomib+Dexamethasone	2	55	NCT01083602	Completed
R/R MM	Panobinostat+Bortezomib	1	62	NCT00532389	Completed
R/R MM	Panobinostat+Carfilzomib	1/2	80	NCT01496118	Completed
R/R MM	Panobinostat+Carfilzomib	1	32	NCT01549431	Completed
R/R MM	Panobinostat+Carfilzomib	1	46	NCT01301807	Active, not recruiting
R/R MM	Panobinostat+Lenalidomide+Dexamethasone	2	32	NCT01651039	Completed
R/R MM	Panobinostat+Bortezomib+Lenalidomide+Dexamethasone	1	21	NCT01965353	Completed
R/R MM	Panobinostat+Daratumumab+Bortezomib+Dexamethasone	1	27	NCT04956302	Recruiting
Relapsed MM	Panobinostat+Melphalan	1/2	40	NCT00743288	Completed
Adult MM	Panobinostat+Lenalidomide+Dexamethasone	1	46	NCT00532675	Completed
ND transplant-eligible MM	Panobinostat+Bortezomib+Lenalidomide+Dexamethasone	1	77	NCT01440582	Completed
R/R MM before autoSCT	Panobinostat+Gemcitabine+Busulfan+Melphalan	2	83	NCT02506959	Active, not recruiting
MM after autoSCT	Panobinostat	2	30	NCT02722941	Active, not recruiting
Recurrent MM, lymphoma	Panobinostat+Everolimus	1/2	124	NCT00918333	Completed
R/R MM, lymphoma	Panobinostat+Everolimus	1	11	NCT00962507	Completed
Refractory CTCL	Panobinostat	2	139	NCT00425555	Completed
R/R PTCL, ENKT	Panobinostat+Bortezomib	2	25	NCT00901147	Completed
R/R MCL	Panobinostat+Bortezomib	1	3	NCT01504776	Completed
R/R DLBCL	Panobinostat	2	35	NCT01523834	Completed
R/R WM	Panobinostat	2	39	NCT00936611	Completed
R/R NHL	Panobinostat	2	41	NCT01261247	Completed
R/R classical HL	Panobinostat	2	129	NCT00742027	Completed
R/R HL	Panobinostat+Lenalidomide	2	24	NCT01460940	Completed
Relapsed HL	Panobinostat/+Ifosfamide+Carboplatin+Etoposide	1/2	62	NCT01169636	Completed
HL maintenance	Panobinostat	3	41	NCT01034163	Completed
R/R lymphoma	Panobinostat+Everolimus	1/2	31	NCT00967044	Completed
Pediatric lymphoma, AML, ALL	Panobinostat for lymphoma, Panobinostat+Cytarabine for Leukemia	1	30	NCT01321346	Completed
High-risk AML, MDS after HSCT	Panobinostat	3	52	NCT04326764	Active, not recruiting
AML younger than 65 years old	Panobinostat+Chemotherapy	1	40	NCT01242774	Completed
ND AML older than 65 years old	Panobinostat+Idarubicin+Cytarabine	1/2	46	NCT00840346	Completed
ND AML, advanced MDS	Panobinostat+Daunorubicin+Cytarabine	1	29	NCT01463046	Completed
Refractory AML	Panobinostat	2	59	NCT00880269	Completed
R/R AML	Panobinostat+Azacitidine+Mitoxantrone	1	59	NCT01055483	Completed
AML, MDS	Panobinostat+Decitabine	1/2	52	NCT00691938	Completed
AML, MDS, CMML	Panobinostat+Azacitidine	1	10	NCT01613976	Completed
AML, MDS, CMML	Panobinostat+Azacitidine	1/2	113	NCT00946647	Completed
Previously treated chronic phase CML	Panobinostat+Imatinib	1	9	NCT00686218	Completed
MF	Panobinostat+Ruxolitinib	1	61	NCT01433445	Completed
MF	Panobinostat+Ruxolitinib	1/2	20	NCT01693601	Completed
MF, PV, GVHD, AML	Panobinostat/+Ruxolitinib	4	298	NCT02386800	Recruiting
Advanced hematologic malignancies	Panobinostat	1/2	175	NCT00621244	Completed
AlloSCT	Panobinostat+Sirolimus+Tacrolimus	2	42	NCT02588339	Completed
AlloSCT	Panobinostat+Corticosteroids	1/2	22	NCT01111526	Completed
Chidamide					
ND PTCL	Chidamide+CHOP	1	30	NCT02809573	Completed
ND PTCL	Chidamide+CHOEP	1/2	100	NCT02987244	Recruiting
ND PTCL	Chidamide+Azacitidine/+CHOP	3	107	NCT05075460	Not yet recruiting
ND PTCL unfit for conventional chemotherapy	Chidamide+Azacitidine	2	28	NCT04480125	Recruiting
R/R PTCL	Chidamide+Parsaclisib	1/2	28	NCT05083208	Not yet recruiting
R/R PTCL	Chidamide+Lenalidomide	2	44	NCT04329130	Recruiting
R/R PTCL	Chidamide+Sintilimab	2	51	NCT04512534	Recruiting
R/R PTCL	Chidamide+Sintilimab+Azacitidine	2	30	NCT04052659	Not yet recruiting
R/R PTCL	Chidamide v.s. Mitoxantrone Hydrochloride Liposome Injection	3	190	NCT04668690	Not yet recruiting
ND AITL	Chidamide+CHOP	2	23	NCT03853044	Active, not recruiting
R/R ATIL	Chidamide+Azacitidine	2	20	NCT05179213	Not yet recruiting
R/R AITL	Chidamide+Sintilimab	2	83	NCT04831710	Not yet recruiting
R/R AITL	Chidamide+Rituximab+Lenalidomide	NA	26	NCT04319601	Recruiting
ND ENKT	Chidamide+Sintilimab	2	30	NCT04994210	Recruiting
R/R ENKT	Chidamide+Sintilimab	1/2	40	NCT03820596	Completed
Stage I/II ENKT	Chidamide/+Gemcitabine+Cisplatin+Dexamethasone+Radiotherapy	NA	76	NCT04511351	Recruiting
R/R CTCL	Chidamide+Sintilimab	2	52	NCT04296786	Recruiting
T-NHL before autoSCT	Chidamide+Carmustine+Etoposide+Cytarabine+Melphalan	2	23	NCT05367856	Not yet recruiting
ND double-expressor DLBCL	Chidamide/+RCHOP	3	418	NCT04231448	Recruiting
R/R DLBCL	Chidamide+Rituximab+Anti-PD-1 Antibody	2	27	NCT05115409	Not yet recruiting
R/R transplant-ineligible DLBCL	Chidamide+Rituximab+Gemcitabine+Oxaliplatin	2	54	NCT04022005	Recruiting
R/R B-NHL	Chidamide	2	100	NCT03245905	Recruiting
R/R B-NHL before CAR-T	Chidamide/+Fludarabine+Cyclophosphamide	1/2	120	NCT05370547	Recruiting
Aggressive R/R B-NHL undergoing decitabine-primed tandem CD19/CD20 CAR-T	Chidamide v.s. Decitabine v.s. Chidamide+Decitabine	1/2	80	NCT04553393	Recruiting
R/R NHL	Chidamide+Chiauranib	1/2	9	NCT03974243	Completed
R/R NHL	Chidamide+Decitabine+Immune checkpoint inhibitors	1/2	100	NCT05320640	Recruiting
NHL who relapsed after CAR-T	Chidamide+Decitabine	1/2	100	NCT04337606	Recruiting
NHL	Chidamide	1	13	NCT02697552	Completed
ND primary central nervous system lymphoma	Chidamide+Rituximab+Methotrexate	2	51	NCT04516655	Not yet recruiting
R/R classical HL	Chidamide+Decitabine+Camrelizumab	2	100	NCT04233294	Recruiting
Anti-PD-1 antibody-resistant classical HL	Chidamide/+Decitabine+Camrelizumab	2	200	NCT04514081	Recruiting
Aggressive lymphoma	Chidamide+BEAC+autoSCT	2	69	NCT03629873	Active, not recruiting
R/R MM	Chidamide+Lenalidomide+Dexamethasone	2	25	NCT03605056	Not yet recruiting
Primary high-risk MM	Chidamide/+Bortezomib+Lenalidomide+Dexamethasone	1/2	50	NCT04025450	Recruiting
CBF AML	Chidamide v.s. Cytarabine	1/2	250	NCT03031262	Recruiting
R/R AML	Chidamide+Azacitidine+Venetoclax	2	30	NCT05305859	Not yet recruiting
R/R AML	Chidamide+Cladribine	2	31	NCT05330364	Recruiting
R/R AML	Chidamide+Decitabine+Priming IAG	2	40	NCT03985007	Completed
R/R AML	Chidamide/+Azacitidine+HAG	2	210	NCT05029141	Recruiting
AML after transplant	Chidamide+Azacitidine	1/2	20	NCT05270200	Recruiting
T-ALL	Chidamide+Ruxolitinib	1/2	50	NCT05075681	Recruiting
B-ALL undergoing haploidentical alloSCT	Chidamide+Ruxolitinib	2	50	NCT05088226	Recruiting
Adult Ph-like ALL	Chidamide v.s. Dasatinib	2/3	120	NCT03564470	Recruiting
HLH	Chidamide+VP-16+Methylprednisolone	NA	20	NCT05137522	Recruiting
ENKT-HLH	Chidamide+Sintilimab v.s Azacitidine+Sintilimab v.s. L-DEP	2	37	NCT05008666	Not yet recruiting
Steroid-resistant/steroid-dependent Severe cGVHD	Chidamide	1/2	20	NCT05140616	Recruiting
Romidepsin					
**Conditions**	**Interventions**	**Phase**	**Patients**	**NCT number**	**Status**
ND PTCL	Romidepsin/+CHOP	3	421	NCT01796002	Active, not recruiting
ND PTCL	Romidepsin+Lenalidomide	2	35	NCT02232516	Active, not recruiting
ND PTCL	Romidepsin+CHOP	1/2	37	NCT01280526	Completed
ND young nodal PTCL before HSCT	Romidepsin+CHOEP	1/2	89	NCT02223208	Active, not recruiting
R/R PTCL	Romidepsin+Ixazomib	1/2	11	NCT03547700	Active, not recruiting
R/R PTCL	Romidepsin+Carfilzomib	1/2	50	NCT03141203	Completed
R/R PTCL	Romidepsin+Gemcitabine	2	20	NCT01822886	Completed
R/R PTCL	Romidepsin+Pembrolizumab	1/2	39	NCT03278782	Recruiting
R/R PTCL	Romidepsin+Ifosfamide+Carboplatin+Etoposide	1	22	NCT01590732	Completed
Progressive or relapsed PTCL	Romidepsin	2	131	NCT00426764	Completed
Progressive or relapsed PTCL	Romidepsin	1/2	51	NCT01456039	Completed
PTCL, CTCL	Romidepsin	2	131	NCT00007345	Completed
R/R mature TCL	Romidepsin+Venetoclax	2	9	NCT03534180	Active, not recruiting
R/R TCL	Romidepsin+Tenalisib	1/2	33	NCT03770000	Completed
R/R TCL	Romidepsin+Parsaclisib	1	20	NCT04774068	Recruiting
R/R TCL	Romidepsin+Duvelisib v.s. Bortezomib+Duvelisib	1	114	NCT02783625	Active, not recruiting
R/R TCL	Romidepsin+Azacitidine+Lenalidomide+Dexamethasone	1	30	NCT04447027	Recruiting
R/R AITL	Romidepsin+Sintilimab	2	83	NCT04831710	Not yet recruiting
R/R ENKT	Romidepsin	1	16	NCT01913119	Completed
TCL	Romidepsin+Azacitidine+Duvelisib	1	60	NCT04639843	Not yet recruiting
TCL, T-ALL after alloSCT	Romidepsin	1	10	NCT02512497	Recruiting
Previously treated PTCL, DLBCL	Romidepsin+Gemcitabine+Dexamethasone+Cisplatin	1	21	NCT01846390	Completed
CTCL	Romidepsin+Brentuximab Vedotin	1	27	NCT02616965	Recruiting
R/R CTCL	Romidepsin+doxorubicin HCl liposomal	1	24	NCT01902225	Completed
R/R CTCL	Romidepsin	2	102	NCT00106431	Completed
T-NHL after autoSCT	Romidepsin maintenance	2	47	NCT01908777	Active, not recruiting
R/R NHL	Romidepsin	2	35	NCT00077194	Completed
R/R NHL	Romidepsin+Alisertib	1	26	NCT01897012	Completed
R/R NHL	Romidepsin+Carfilzomib+Lenalidomide	1/2	31	NCT02341014	Active, not recruiting
R/R aggressive lymphoma	Romidepsin+Gemcitabine+Oxaliplatin+Dexamethasone	1	24	NCT02181218	Completed
R/R lymphoma, myeloma	Romidepsin+Lenalidomide	1/2	62	NCT01755975	Active, not recruiting
R/R MM	Romidepsin	2	50	NCT00066638	Completed
R/R AML	Romidepsin	2	47	NCT00062075	Completed
R/R leukemia, MDS, MPN	Romidepsin+Decitabine	1	36	NCT00114257	Completed
R/R CLL/SLL	Romidepsin+Bortezomib	1	18	NCT00963274	Completed

*AITL* angioimmunoblastic T-cell lymphoma, *ALL* acute lymphoblastic leukemia, *alloSCT* allogenic stem cell transplantation, *AML* acute myeloid leukemia, *ASCT* autologous stem cell transplantation, *BL* Burkitt’s lymphoma, *CLL* chronic lymphocytic leukemia, *CML* chronic myeloid leukemia, *CTCL* cutaneous T-cell lymphoma/leukemia, *DLBCL* diffuse large B-cell lymphoma, *ENKT* extranodal NK/T-cell lymphoma, *FL* follicular lymphoma, *GVHD* graft-versus-host disease, *HL* Hodgkin lymphoma, *ITD* internal tandem duplication, *MCL* mantle cell lymphoma, *MDS* myelodysplastic syndrome, *MF* myelofibrosis, *MM* multiple myeloma, *ND* newly diagnosed, *NHL* non-Hodgkin lymphoma, *PV* polycythemia vera, *PTCL* peripheral T-cell lymphoma, *R* rituximab, *R/R* relapsed/refractory, *SLL* small lymphocytic lymphoma

In the benzamide group, chidamide, also named CS055 or tucidinostat, inhibits HDAC1, HDAC2, HDAC3, and HDAC10, and has been approved by the China NMPA and EMA to treat R/R PTCL.^674,675^ For R/R PTCL patients, the ORR of chidamide was 28%, and the median OS was 21.4 months, with good tolerance in clinical trials.^676^ In a real-world study, the ORR of chidamide monotherapy was 39.06%, and that of chidamide combined with chemotherapy was 51.18% in R/R PTCL.^677^ In addition, there are numerous ongoing clinical trials exploring its efficacy in NHL (mostly T-cell lymphoma), AML, ALL, MM, and hemophagocytic lymphohistiocytosis.^678^ In addition to the approved chidamide, other benzamide-based HDAC inhibitors are being explored in clinical trials. Entinostat, also known as MS275, inhibits HDAC1, HDAC3, and HDAC8. Preclinical studies revealed that entinostat relieved the epigenetic silencing of LAT2 caused by AML1/ETO, and inhibited leukemic maintenance.^679–681^ Entinostat stimulated apoptosis alone and synergized with rituximab in B-cell lymphoma, with BCL2 inhibitors in HL, and with bendamustine in MM.^682–684^ Various clinical trials are investigating its efficacy in MDS, AML, CMML, and lymphoma. However, bendamustine combined with azacytidine displayed worse ORR and OS in AML and MDS compared with azacytidine treatment alone, indicating antagonism and higher toxicity of the two agents in this setting.^685,686^ Mocetinostat, also named MGCD0103, inhibits HDAC1, HDAC2, HDAC3, and HDAC11. Mocetinostat triggered H3 and H4 acetylation and exerted significant antitumor effects in various cancer cells.^351^ Various clinical trials have explored its effect on R/R lymphoma and AML. Domatinostat, also named 4SC-202, inhibits HDAC1, HDAC2, and HDAC3, and displayed a manageable safety profile with signs of antitumor effects in a phase I trial enrolling patients with advanced hematological malignancies.^687^ Tacedinaline, also named CI-994, is a selective HDAC1 inhibitor and has been explored in advanced MM patients.

In the cyclic peptide group, romidepsin, also named FK228 or depsipeptide, inhibits HDAC1 and HDAC2 and was the second HDAC inhibitor approved by the U.S. FDA to treat CTCL and PTCL. Romidepsin inhibited proliferation and angiogenesis and promoted cell cycle arrest and apoptosis in various cancer cells.^688^ For previously treated or refractory CTCL patients, romidepsin monotherapy displayed an ORR of 34%.^689,690^ For R/R PTCL patients, romidepsin monotherapy displayed an ORR of 38%.^691^ Numerous ongoing clinical trials are investigating romidepsin combined with anti-PD-1 monoclonal antibody, PIs, PI3K inhibitors, BCL2 inhibitors, IMiDs, and chemotherapy in R/R T-cell NHL.

#### Dual-target HDAC inhibitors

In addition to the classic HDAC inhibitors, dual-target HDAC inhibitors have also demonstrated favorable antitumor effects in preclinical models. CUDC-101, an HDAC I/receptor tyrosine kinase (RTK) bifunctional inhibitor, inhibits HDAC I, EGFR, and HER2 and has been demonstrated effective in preclinical models of gemcitabine-treated lymphoma, APL, and several solid tumors.^692,693^ Several clinical trials have investigated its role in solid tumors, while no trials on hematologic malignancies have been conducted. An HDAC6/JAK2 inhibitor was designed based on vorinostat and pacritinib, and showed preclinical efficacy in AML and ALL.^694^ CUDC-907, an HDAC/PI3K inhibitor, exerted an ORR of 37% in R/R DLBCL patients, including those with MYC-alterations.^695^ HDAC/LSD1 inhibitors and HDAC I/tubulin inhibitors display favorable efficacy in solid tumors, such as colon cancer and breast cancer, but few studies have explored their application to hematologic malignancies.^696,697^

#### PROTACs for HDACs

The reversibility of classic small-molecule HDAC inhibitors requires sustainable exposure to a high in vivo drug concentration to maintain sufficient inhibition. However, maintaining a high in vivo drug concentration is sometimes challenging. Proteolysis-targeting chimeras (PROTACs) provide an innovative strategy for degrading HDACs more persistently. A PROTAC is a small bifunctional compound consisting of a ligand for the target protein, an E3 ligase recognition moiety, and a linker. After the ligand binds with the target protein on one side, the E3 ligase on the other side mediates the ubiquitination of the target protein by the ubiquitin-conjugating E2 enzyme. The target protein labeled with ubiquitin is recognized and degraded by proteasomes. The ubiquitination and degradation process are highly efficient and recyclable, making PROTACs a promising strategy to degrade HDACs. Several HDAC-targeted PROTACs have been developed in recent years.

According to the E3 ligase complex, these PROTACs are mainly divided into cereblon (CRBN)-based PROTACs and Von Hippel-Lindau (VHL)-based PROTACs. The first-in-class HDAC6-targeted PROTAC, compound 9c, was developed by conjugating a pan-HDAC nonselective inhibitor to thalidomide as CRBN. The concentration of half-maximal degradation (DC_50_) for HDAC6 was 34 nM, and HDAC6 was significantly degraded by 9c at a concentration of 80 nM in multiple myeloma MM.1S cells. However, other HDACs were also inhibited by 9c, indicating the need for HDAC6-targeted PROTACs with higher selectivity and specificity.^698^ Later, HDAC6-targeted PROTACs with higher selectivity were developed by conjugating a selective HDAC6 inhibitor, nexturastat A, to pomalidomide as CRBN. The degrader, NP8, exerted efficient HDAC6 degradation in MM.1S cells with a DC_50_ of 3.8 nM without affecting other types of HDACs.^699^ Another degrader, NH2, exerted similarly efficient HDAC6 degradation in MM.1S cells with a DC_50_ of 3.2 nM without affecting other types of HDACs. Moreover, the onset of degradation occurred within an hour and peaked at 6–8 h, and HDAC6 rapidly recovered three hours after washout, indicating its efficient and reversible capability to degrade HDAC6.^700^

In addition to CRBN-based PROTACs, VHL-based PROTACs targeting HDAC have also been developed. An HDAC6 degrader, compound 3j, was designed by conjugating nexturastat A to VHL, which showed robust HDAC6 degradation with a DC_50_ of 7.1 nM in MM.1S cells. Furthermore, the maximal degradation (D_max_) of HDAC6 reached up to 90% in MM.1S cells.^701^ An HDAC3 degrader, XZ9002, was developed by conjugating a selective HDAC I inhibitor, SR-5228, to VHL. The DC_50_ of HDAC3 in breast cancer MDA-MB-468 cells was 42 nM.^702^

#### Combination treatment of HDAC inhibitors with HMAs

Inspired by the encouraging efficacy of HMAs in treating leukemia, numbers of clinical trials have explored the combination treatment of HMAs and HDAC inhibitors in AML and ALL. The majority of clinical trials focused on AML. However, most results were disappointing. A phase I study explored the combination of the HDAC inhibitor AR-42 with decitabine in 13 newly diagnosed or relapsed/refractory AML patients. The ORR was only 23.1% with dose-limiting toxicities occurring at the third dosage. The results were not satisfying, probably due to the small sample size, high-risk baseline clinical features of participants, and the relatively mild HDAC inhibition capability of AR-42.^624^ A phase I study investigated decitabine combined with the HDAC inhibitor valproic acid or not in 25 AML patients. The ORR of decitabine monotherapy group was similar to that in combination treatment group. The addition of valproic acid to decitabine showed little treatment effect bonus, but caused encephalopathy. The evident effect of valproic acid on central nervous system, as evidenced by its approved indications as seizures, narrowed its application in combination treatment in AML patients.^703^ The addition of valproate to decitabine in AML treatment was proved insignificant again in a phase II trial with larger sample size.^704^ Moreover, the combination treatment of decitabine even with the commonly recognized effective HDAC inhibitor vorinostat still exerted limited ORR (23%) in AML patients.^705^ In relapsed ALL, combined treatment of vorinostat with decitabine exerted an ORR of 39–46.2%, which was higher than that in AML. However, treatment-related infections were common.^706,707^ The failure of combination treatment of HDAC inhibitors with HMAs could be attributable to the potential epigenetic antagonism. Several clinical trials are still ongoing in PTCL and DLBCL. Extensive studies are required to thoroughly reveal the exact mechanism and interaction between the two epigenetic therapies.

#### Combination treatment of HDAC inhibitors with immune treatment

HDACs can regulate both innate and adaptive immune response. In innate immune response, HDACs could promote or inhibit Toll-like receptor (TLR) signaling pathways determined by the types of HDACs and diseases, leading to either an increase or decrease in cytokine and chemokine secretion. Regarding adaptive immune response, HDAC inhibitors could upregulate MHC-I and promote antigen processing, which amplified the antitumor reaction.^708^ Panobinostat has been reported to activate Notch pathway and enhance the anti-leukemic influence of human γδT cells in vitro,^709^ and could exert synergistic effect with interferon-α in mouse models.^710^ Trichostatin A, an HDAC inhibitor, has also been proved to induce dendritic cell differentiation from AML leukemic blasts in vitro.^711^ These encouraging preclinical findings indicate the promising application of combination treatment of HDAC inhibitors and immune treatment in hematologic malignancies. The number of clinical trials are investigating the combined treatment of HDAC inhibitors with rituximab in B-cell lymphoma, with lenalidomide in MM, and with brentuximab vedotin in CTCL patients. Various ongoing clinical trials are also exploring the combined treatment of HDAC inhibitors with PD-1 monoclonal antibodies in the treatment of HL, DLBCL, FL, PTCL, CTCL, AITL, ENKT, and HLH secondary to ENKT (Table 4). Their results are widely anticipated, and will better reveal the optimal individualized treatment of hematologic malignancies.

### Targeting other histone modification agents

Although HDAC inhibitors are one of the most explored and applied epigenetic regimens, agents targeting other histone modification agents are also being developed (Fig. 1).

Regarding histone acetylation, agents targeting writers mainly include CBP/P300 inhibitors. CCS1477, a CBP inhibitor, is being explored to treat NHL, MM, AML, and MDS in a phase I/IIa trial (NCT04068597).^712^ Agents targeting readers include BET inhibitors. OTX015 inhibited proliferation and stimulated cell cycle arrest and apoptosis in leukemia cells. It also downregulated BRD2, BRD4, and MYC in vitro.^713^ In lymphoma, OTX015 stimulated the apoptosis of DLBCL cells with mutations in MYD88, CD79B, or CARD11, and repressed MYC- and E2F1-related expression.^714^ OTX015 showed mild efficacy in acute leukemia and lymphoma patients, while no efficacy was found in MM patients.^715^ CPI-0610, GSK525762, RO6870810, and FT-1101 are being explored in hematologic malignancies in clinical trials.^716^ Apart from the classic BET inhibitors, BET-targeted PROTACs have been reported. ARV-825 was developed by conjugating the BRD4 inhibitor OTX015 to pomalidomide as CRBN. ARV0825 exerted rapid, efficient and sustained degradation of BRD4 and lethal activity compared to BET inhibitors in Burkitt lymphoma cells, mantle cell lymphoma cells, post-MPN secondary AML cells, and T-cell ALL cells.^717–721^ dBET1 was generated by conjugating the BRD4 inhibitor, JQ1, to phthalimide as CRBN. The efficient and specific degradation of BRD2, BRD3, and BRD4 was accompanied by superior apoptosis induction in AML cells and xenograft models.^722^

For histone methylation, agents targeting writers include EZH2 inhibitors and DOT1L inhibitors. EZH2 inhibitors in the clinical stage include tazemetostat, CPI-1205, SHR2554, and PF-06821497. Tazemetostat has been approved by the U.S. FDA to treat metastatic or advanced epithelioid sarcoma. Although most of the EZH2 inhibitor clinical trials focus on solid tumors, several trials are investigating its effect on R/R B-NHL.^723^ The DOT1L inhibitor, pinometostat, also named EPZ5676, has been demonstrated to impair H3K79 methylation and moderately effective in treating *MLL*-rearranged leukemia.^724^ Agents targeting histone methylation erasers mainly include LSD1 inhibitors, also named KDM1A inhibitors. ORY-1001, TCP, and GSK2879552 are being investigated in the treatment of R/R AML in clinical trials. INCB059872 and IMG-7289 have been explored in MPN trials. CC-90011 was investigated in R/R NHL.^725^

In terms of histone phosphorylation, the JAK2 inhibitors (ruxolitinib, fedratinib, and pacritinib) have been approved by the U.S FDA to treat MPN.^726^ Various clinical trials have been investigating their role in CMML, CLL, ALL, and post-MPN AML.^727,728^ In addition, aurora inhibitors have been developed and have shown synergism with docetaxel in apoptosis stimulation to inhibit lymphoma.^729,730^

## Conclusion

In conclusion, epigenetic regulation plays a fundamental role in hematopoiesis and oncogenesis of hematologic malignancies. Abnormal DNA methylation profiles, including genome-wide hypomethylation and aberrant hypermethylation or hypomethylation of CpG islands, are frequently observed in hematologic malignancies. Upregulation or mutations of DNA methylation writers (DNMT1, DNMT3A, and DNMT3B) are pathogenic in AML. Readers of DNA methylation mainly consist of MBD-containing proteins, methyl-CpG binding zing fingers, and SRA domain-containing proteins, whose upregulation is frequently identified in CML, NHL, and AML, respectively. DNA methylation erasers mainly include the TET family, whose mutations, translocation, and upregulation are relatively common in AML. Correspondingly, HMAs have been developed to target aberrant DNA methylation profiles and have been demonstrated effective to treat MDS, AML, and CMML. Furthermore, histone acetylation and methylation are involved in hematologic oncogenesis. KATs are histone acetylation writers, whose mutations are detected in DLBCL and translocations are identified in AML. HDACs are histone acetylation erasers, whose aberrant expression has been demonstrated in lymphoma. Correspondingly, HDAC inhibitors have been proposed and demonstrated effective in treatment of CTCL, PTCL, and MM. Mutations, translocations, and aberrant expression of histone methylation writers, KMTs, and histone methylation erasers, KDMs, are also found in AML and other hematologic malignancies. Dysregulation of miRNAs and lncRNAs also contributes to hematologic oncogenesis. In addition to DNMT inhibitors and HDAC inhibitors, innovative epigenetic treatment targeting KATs, BETs, KMTs, KDMs, and ncRNAs are emerging and will provide novel treatment strategies in hematologic malignancies.

However, the relationship of cancer epigenetics with interdependent mechanisms like cancer immunology or metabolism should be explored further. In the future, more accurate diagnostic and prognostic information detected by high-throughput genomic technologies can provide more precise and individualized therapeutic options. Moreover, the ncRNA could possibly serve as diagnostic and prognostic molecular biomarkers in the future clinical settings. With the rapid growth of associated scientific research methods and technologies in this academic sector, ncRNA-targeted therapy may soon be a realistic treatment option for patients with hematologic malignancies.

## Supplementary information

Supplementary Table 1
