# Supplementary material for: Epigenetic regulation in hematopoiesis and its implications in the targeted therapy of hematologic malignancies
Source: Signal Transduct Target Ther. 2023 Feb 17;8:71. doi: 10.1038/s41392-023-01342-6 (PMC9935927; doi:10.1038/s41392-023-01342-6)
Supplement: Supplementary file 1 — Supplementary Table 1 [file 41392_2023_1342_MOESM1_ESM.docx]

Supplementary Materials for

Epigenetic regulation in hematopoiesis and its implications in the targeted therapy of hematologic malignancies

Ailin Zhao^1^, Hui Zhou^1^, Jinrong Yang^1^, Meng Li, Ting Niu^*^.

Correspondence to: [niuting@wchscu.cn](mailto:xxxxx@xxxx.xxx)

**This PDF file includes:**

Tables S1

Table S1.

The clinical trials of IDH inhibitors in combination with other therapies.

| NCT Number | Conditions | Interventions | Phase | Number Enrolled |
| --- | --- | --- | --- | --- |
| Ivosidenib/Enasidenib | | | | |
| NCT04493164 | AML, high-risk MDS, MPN | Ivosidenib, liposome-encapsulated daunorubicin-cytarabine | Phase 2 | 30 |
| NCT03471260 | AML, hematopoietic and lymphoid system neoplasm, MDS | Azacitidine, ivosidenib, venetoclax | Phase 1/2 | 30 |
| NCT03173248 | AML, MDS | Ivosidenib with azacitidine | Phase 3 | 148 |
| NCT04774393 | AML, R/R AML | Decitabine and cedazuridine, enasidenib, ivosidenib, venetoclax | Phase 1/2 | 84 |
| NCT04250051 | R/R AML, R/R MDS | Ivosidenib and combination chemotherapy | Phase 1 | 25 |
| NCT04044209 | AML /MDS | Ivosidenib, nivolumab | Phase 2 | Withdrawn |
| NCT02632708 | AML /MDS | Ivosidenib, enasidenib, cytarabine | Phase 1 | 153 |
| NCT05010772 | AML | Decitabine and cedazuridine, enasidenib, gilteritinib | Phase 1 | 125 |
| NCT05401097 | AML | Azacitidine, enasidenib | Phase 2 | 125 |
| NCT02677922 | AML | Ivosidenib, azacitidine, enasidenib | Phase 1/2 | 130 |
| NCT04655391 | R/R AML | Decitabine, enasidenib | Phase 1 | Withdrawn |
| NCT04603001 | AML, MDS, CMML, MPN | LY3410738(IDH1/2 inhibitor), venetoclax, azacitidine | Phase 1 | 260 |
| NCT05441514 | R/R AML | Cobimetinib, enasidenib | Phase 1 | 15 |
| NCT03683433 | Acute bilineal leukemia, acute biphenotypic leukemia, CML | Azacitidine, enasidenib | Phase 2 | 50 |
| NCT04281498 | Accelerated/blast-phase MPN, chronic-phase myelofibrosis | Ruxolitinib, enasidenib | Phase 2 | 32 |
| NCT04092179 | AML | Enasidenib, venetoclax | Phase 1/2 | 48 |
| NCT03825796 | R/R AML | Enasidenib, liposome-encapsulated daunorubicin-cytarabine | Phase 2 | 18 |
| NCT02577406 | AML | Enasidenib, BSC, azacitidine | Phase 3 | 319 |
| NCT03383575 | AML, CML | Azacitidine, enasidenib | Phase 2 | 105 |
| NCT03013998 | AML | Samalizumab (BAML-16-001-S1), BI 836858 (BAML-16-001-S2) | Phase 1/2 | 2000 |
| NCT04075747 | AML | CPX-351, venetoclax, midostaurin, enasidenib | Phase 1 | 57 |
| Olutasidenib | | | | |
| NCT02719574 | AML, MDS | Olutasidenib, azacitidine, cytarabine | Phase 1/2 | 336 |
